# Interaction of BACH2 with FUS promotes malignant progression of glioma cells via the TSLNC8–miR‐10b‐5p–WWC3 pathway

**DOI:** 10.1002/1878-0261.12795

**Published:** 2020-09-26

**Authors:** Yang Yang, Xiaobai Liu, Jian Zheng, Yixue Xue, Libo Liu, Jun Ma, Ping Wang, Chunqing Yang, Di Wang, Lianqi Shao, Xuelei Ruan, Yunhui Liu

**Affiliations:** ^1^ Department of Neurosurgery Shengjing Hospital of China Medical University Shenyang China; ^2^ Liaoning Clinical Medical Research Center in Nervous System Disease Shenyang China; ^3^ Key Laboratory of Neuro‐oncology in Liaoning Province Shenyang China; ^4^ Department of Neurobiology School of Life Sciences China Medical University Shenyang China; ^5^ Key Laboratory of Cell Biology Ministry of Public Health of China China Medical University Shenyang China; ^6^ Key Laboratory of Medical Cell Biology Ministry of Education of China China Medical University Shenyang China

**Keywords:** BACH2, FUS, glioma, miR‐10b‐5p, TSLNC8, WWC3

## Abstract

Glioma, a common malignant tumour of the human central nervous system, has poor prognosis and limited treatment options. Dissecting the biological mechanisms underlying glioma pathogenesis can facilitate the development of better therapies. Here, we investigated the endogenous expression of BTB and CNC homolog 2 (BACH2), fused in sarcoma (FUS), TSLNC8 and microRNA (miR)‐10b‐5p in glioma cells and tissues. We studied the interaction between BACH2 and FUS and its contribution to glioma progression. We demonstrated that the interaction between BACH2 and FUS promoted glioma progression via transcriptional inhibition of TSLNC8. Overexpression of TSLNC8 restrained glioma progression by suppressing miR‐10b‐5p. Binding of TSLNC8 to miR‐10b‐5p attenuated the suppression of WWC family member 3 (WWC3) by miR‐10b‐5p and activated the Hippo signalling pathway. Growth of subcutaneous xenografts could be inhibited by knockdown of BACH2 or FUS, by overexpressing TSLNC8 or a combination of the three, also leading to a prolonged survival in nude mice. Our results indicate that the BACH2 and FUS/TSLNC8/miR‐10b‐5p/WWC3 axis is responsible for glioma development and could serve as a potential target for the development of new glioma therapies.

## Graphical abstract

Interaction of BTB and CNC homolog 2 (BACH2) with fused in sarcoma (FUS) promoted glioma progression via transcriptional inhibition of TSLNC8. Overexpression of TSLNC8 restrained glioma progression by suppressing miR‐10b‐5p. Binding of TSLNC8 to miR‐10b‐5p attenuated the suppression of WWC family member 3 (WWC3) by miR‐10b‐5p and activated the Hippo signalling pathway. BACH2 and FUS/TSLNC8/miR‐10b‐5p/WWC3 axis is responsible for glioma development and could serve as a potential target for glioma therapies.

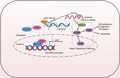

**Abbreviations**
BACH2: BTB and CNC homolog 2
CCK‐8: cell counting kit‐8
Co‐IP: co‐immunoprecipitation
FUS: fused in sarcoma
GAPDH: glyceraldehyde‐3‐phosphate dehydrogenase
HA: human astrocyte
HEK‐293T: human embryonal kidney 293T cell, ChIP, chromatin immunoprecipitation
lncRNAs: long noncoding RNAs
miRNAs: microRNAs
NBTs: normal brain tissues
NC: negative control
qRT–PCR (qPCR): real‐time quantitative polymerase chain reaction
RIP: RNA‐binding protein immunoprecipitation
RISC: RNA‐induced silencing complex
shRNA: short hairpin RNA
TSS: transcription start sites
WWC3: WWC family member 3
YAP: Yes‐activated protein

## 1 Introduction

Glioma is the most common malignant tumour in the human central nervous system [1]. Glioma accounts for > 70% of all brain tumours, of which glioblastoma constitutes 65%, with a higher degree of malignancy [2]. Although several therapies are currently available, including surgery, radiotherapy, chemotherapy and biotherapy, they have a low survival rate. The average median survival time for glioma is only 12–18 months [3]. Therefore, understanding the biological behaviour and mechanism underlying glioma is key to improve its prognosis and for the development of new therapies.

BTB and CNC homology 2 (BACH2) is a transcription factor with a basic region leucine zipper (bZip) involved in immune system regulation. The inhibition of BACH2 in activated B cells induces the differentiation of plasma cells into immunoglobulin M‐secreting cells [4]. BACH2 is a B‐cell‐specific transcriptional inhibitor that acts as a tumour suppressor in B‐cell malignancy [5]. BACH2 is upregulated in tumours of patients with PD‐1 response‐responsive renal cell carcinoma [6]. Furthermore, BACH2 promotes immunosuppression in mouse melanoma xenografts [7]. However, the expression of BACH2 in gliomas has not been well‐studied.

Fused in sarcoma (FUS) is an RNA‐binding protein that is mainly localised in the nucleus and plays a crucial role in RNA transcription. It also plays a role in DNA repair regulation, transcription, splicing, RNA transport and local translation [8]. The overexpression of FUS promotes malignant progression of non‐small cell lung cancer [9] and cervical cancer [10]. A recent study showed that FUS plays a crucial role in regulating angiogenesis in gliomas [11]. However, to the best of our knowledge, no reports are currently available on the biological behaviour or mechanisms underlying glioma.

Long noncoding RNAs (lncRNAs) are 200‐nucleotide‐long endogenous RNAs without protein‐coding functions [12] and play a crucial regulatory role in tumour development. For example, MEG3 promotes autophagy and inhibits proliferation and migration of glioma cells [13], and HOXA10‐AS promotes the growth and survival of glioma cells [14]. It has been reported that TSLNC8 acts as a tumour suppressor and is involved in the regulation of various malignant tumour diseases, including hepatocellular carcinoma [15], breast cancer [16] and non‐small cell lung cancer [17]. Additionally, existing studies have shown that TSLNC8 suppresses cell proliferation and metastasis and promotes cell apoptosis in human glioma [18]. However, the detailed function of TSLNC8 in glioma remains largely unknown. microRNA(miR)‐10b‐5p is located on chromosome 2 and regulates tumour development. Studies have shown that high levels of miR‐10b‐5p expression in patients with breast cancer are related to poor prognosis [19]. The overexpression of miR‐10b‐5p has been found to promote glioma cell proliferation and inhibit apoptosis [20].

The Hippo signalling pathway regulates stem cell function and plays an important role in tumorigenesis [21]. The WWC protein family (WWC1, WWC2 and WWC3) functions upstream of the Hippo signalling pathway [22]. Recent studies have demonstrated that WWC3 regulates the Wnt and Hippo pathways via Dvl protein and LATS1 to inhibit the invasion and metastasis of lung cancer cells [23]. Although the expression of WWC3 is known to be downregulated in human glioma tissues and cells [24], the mechanism through which WWC3 affects glioma cell behaviour remains to be fully elucidated.

The present study investigated the expression of endogenous BACH2, FUS, TSLNC8 and miR‐10b‐5p in glioma tissues and cells. Furthermore, the effect of these interactions on glioma cell behaviour was determined. The main aim of this study was to elucidate the mechanisms underlying the development of gliomas, thus providing a basis for the development of novel strategies for glioma therapy.

## 2 Materials and methods

### 2.1 Human tissue specimens and cell cultures

Human glioma tissues and normal brain tissues (NBTs) were collected from patients undergoing surgery in the Neurosurgery Department of Shengjing Hospital of China Medical University. Informed consent was obtained before operation. This study was approved by the Ethics Committee of Shengjing Hospital of China Medical University. NBTs collected from fresh autopsy materials of patients who were organs donor or individuals who had died in traumatic events without brain disease were used as negative controls (NC) (*n* = 12). According to the World Health Organization 2007 classification, the glioma tissues were divided into a grade I–II group (*n* = 12) and a grade III–IV group (*n* = 12). The clinical information of donors and patients is provided in Table S1.

Human astrocyte (HA) cells, human glioma cell lines (U87 and U251) and human embryonic kidney cell line (HEK293T) were purchased and cultured as previously described [25].

### 2.2 Quantitative real‐time PCR and reverse transcription

Total RNA was extracted with TRIzol reagent (Life Technologies, Carlsbad, CA, USA). The RNA concentration and quality were determined at a 260/280‐nm ratio using a NanoDrop 2000 Spectrophotometer (Thermo Fisher Scientific, Wilmington, DE, USA). The expression of BACH2, FUS, TSLNC8 and WWC3 mRNA was detected using the One‐Step TB Green™ PrimeScript™ RT‐PCR Kit (Perfect Real Time) (Takara, Shiga, Japan) and a 7500 Fast RT‐PCR System (Applied Biosystems, Waltham, MA, USA). miR‐10b‐5p was reverse‐transcribed using miRNA First‐Strand cDNA Synthesis (Tailing Reaction) (Sangon Biotech, Shanghai, China), and the expression was detected with 2 × SG Fast qPCR Master Mix (Low Rox) (Sangon Biotech). Endogenous controls [glyceraldehyde‐3‐phosphate dehydrogenase (GAPDH) or U6] were used to normalise the RNA expression. The $2^{‐\Delta \Delta C_{t}}$ method was used to calculate the relative quantification. The primers used in the qRT–PCR are provided in Table S2.

### 2.3 Transfections

BACH2 (sh‐BACH2), FUS (sh‐FUS) or WWC3 (sh‐WWC3) short hairpin RNAs (shRNA), TSLNC8 full‐length (TSLNC8‐OE) plasmids, WWC3 full length with 3′‐UTR (WWC3), WWC3 without 3′‐UTR (WWC3 non‐3′‐UTR) plasmids and their respective NC were purchased from GeneChem (Shanghai, China). miR‐10b‐5p agomir, miR‐10b‐5p antagomir and their respective NCs were purchased from GenePharma (Shanghai, China). Cell transfection and selection were conducted as previously reported [26]. Stable transfection was conducted after the site with the highest knockdown efficiency was selected by quantitative real‐time PCR (qRT‐PCR) after 48 h of transient transfection with the above plasmids in Lipofectamine 3000 reagent, according to the manufacturer’s protocols. Both U87 and U251 cells were seeded in 24‐well plates. Once the cells reached 70–80% confluence, stable transfection was performed. G418 (Sigma‐Aldrich, St Louis, MO, USA) and puromycin (BioFroxx, Einhausen, Germany) were used to select the resistant and stably transfected cell clones. The gene expression levels for transient or stable transfection were detected using qRT‐PCR or western blotting (Fig. S1A‐K).

### 2.4 Cell viability assay

Cell viability was performed using CCK‐8 solution (Beyotime Biotechnology, Jiangsu, China) to assess the cell proliferation ability. The assay was performed as previously reported [27].

### 2.5 Transwell assay

Migration and invasion were detected by Transwell assay using chambers with 8‐μm pore polycarbonate membranes (Corning, Corning, NY, USA), as previously reported [28].

### 2.6 Apoptosis evaluation by flow cytometry

ApoScreen Annexin V Apoptosis Kit‐PE (Southern Biotech, Birmingham, AL, USA) was used to detect cell apoptosis, as previously reported [26].

### 2.7 Western blot analysis

RIPA lysate (Beyotime Biotechnology) and nuclear protein extraction kit (Solarbio, Beijing, China) with PMSF were used to extract the total proteins and nucleus or cytoplasm proteins, according to the manufacturer's instructions. An enhanced bicinchoninic acid Protein Assay Kit (Beyotime Biotechnology) was used to analyse the protein concentrations. The primary antibodies were diluted as follows: BACH2 (1 : 500) (Cell Signaling Technology, Danvers, MA, USA), FUS (1 : 1000) (ProteinTech, Rosemont, IL, USA), WWC3 (1 : 100) (Abcam, Cambridge, UK), Yes‐activated protein (YAP) (1 : 1000) (ProteinTech), p‐YAP (1 : 500) (ABclonal Technology, Wuhan, China), GAPDH (1 : 10 000) (ProteinTech) and Histone H3 (1 : 2000) (ProteinTech). The assays were performed as previously reported [29]. GAPDH or Histone H3 was used as internal controls to calculate the integrated density values.

### 2.8 Co‐immunoprecipitation (Co‐IP) and GST pull‐down assays

The interaction between BACH2 and FUS was examined *in vivo* using a Pierce Co‐Immunoprecipitation (Co‐IP) Kit (Thermo Fisher Scientific), according to the manufacturer's protocols. Coupling resin was incubated at 4 °C overnight with the indicated amounts of antibody. The antibody‐coupling resin complexes were then used to precipitate the cell lysates. Anti‐BACH2 (Cell Signaling Technology) and anti‐FUS (ProteinTech) were used to detect the precipitate. For *in vitro* binding assays, GSH‐agarose beads (Thermo Fisher Scientific) were used to purify the GST or GST‐BACH2 fusion bait protein, and His‐tag purification resin beads (Beyotime Biotechnology) were used to purify the His‐FUS fusion protein. GST protein or GST‐BACH2 fusion protein, which was combined with GSH‐agarose beads, was incubated with His‐FUS fusion protein for 6 h at 4 °C. The resulting bead‒protein‒protein complex was precipitated. Proteins isolated using elution buffer were detected by western blotting using anti‐GST (ProteinTech) and anti‐FUS (ProteinTech).

### 2.9 RNA immunoprecipitation (RIP) assay

Pierce™ Magnetic RNA‐Protein Pull‐Down Kit (Thermo Fisher Scientific) was used in the RIP assay, and the assay was conducted as previously reported [28].

### 2.10 Immunofluorescence

The cells were fixed with 4% paraformaldehyde for 30 min, blocked by 5% BSA for 2 h at room temperature and then stained with the appropriate primary and secondary antibodies. The staining was recorded and merged using Olympus immunofluorescence microscopy (Olympus, Shinjuku, Tokyo, Japan) and DP Manager software (Olympus).

### 2.11 Chromatin immunoprecipitation (ChIP) assay

SimpleChIP^®^ Enzymatic Chromatin IP Kit (Agarose Beads) (Cell Signaling Technology) was used to perform the ChIP assay, according to the manufacturer's instructions. The assay was performed as previously described [30]. DNA was immunoprecipitated using an anti‐BACH2 (1 : 50) (Cell Signaling Technology).

The binding site of BACH2 was 5′‐CCTGCCTCAGCCTC‐3′. Primers were designed based on the sequence with a binding site, and control, as the NC, was designed based on the sequence without binding sites. Immunoprecipitated DNA from anti‐BACH2 was amplified by PCR with primers. The primers for each PCR set were as follows: 5′‐GTGTGCAGTGGTGCAATCTT‐3′ and 5′‐GGTGGAGCCCCATCTCTACT‐3′; control, 5′‐TCTGTGATAAGGGGTGAGATTTT‐3′ and 5′‐GGCCTTCTGCACTTGCTATT‐3′.

For each PCR, the corresponding input was taken in parallel for PCR validation.

### 2.12 lncRNAs and miRNA microarrays

Analysis of Human lncRNA Expression Profile chip was used after samples treated with sh‐NC and sh‐BACH2, and analysis was performed by KangChen Biotech (Shanghai, China) using an Agilent chip platform. 60‐mer oligonucleotide probes and chip designed by Agilent Technologies were used. More than 77 000 lncRNAs could be detected.

After samples treated with EV and TSLNC8‐OE, TaqMan™ Array Human MicroRNA A+B Cards Set v3.0 designed by ABI was used to analyse the miRNA expression profile using the AB 7900 HT 384‐Well System. Samples were labelled by FAM, and 754 human miRNAs could be accurately quantified.

### 2.13 Fluorescence *in situ* hybridisation

The TSLNC8 probe (green‐labelled) (GenePharma) was used to identify the TSLNC8 distribution in HA, U87 and U251 cells using an RNA FISH Kit (Cell Crawler) (GenePharma). The assay was performed according to the manufacturer’s instructions with a probe sequence of 5′‐TGAATGGAGGTGTCATCCTG‐3′.

### 2.14 Luciferase reporter gene assay

The full‐length sequence of TSLNC8, the WWC3 3′‐UTR sequence and the mutant sequences of the mir‐10b‐5p binding site were amplified and constructed into pmirGLO Dual‐luciferase miRNA Target Expression Vectors (GenePharma) and performed as previously reported [31]. HEK293T cells were cotransfected with the abovementioned vectors and agomir‐10b‐5p (or agomir‐10b‐5p‐NC). Luciferase activity was detected using a Promega GloMax Luminometer (Promega, Madison, WI, USA) 48 h after transfection, and the relative luciferase activity was normalised to Renilla luciferase activity before recording.

### 2.15 Tumour xenografts in nude mice

The shRNA interference sequences of BACH2 and FUS were recombined into the lentiviral vector pLenti6/BLOCK‐iT using the BLOCK‐iT™ Lentiviral RNAi Gateway™ Vector Kit (Life Technologies). After the identification was correct, human embryonal kidney 293T cells (HEK‐293T) were transfected with the ViraPower packaging mixture. After 48h, the virus supernatant was collected, concentrated and tittered obtaining recombinant lentiviruses Lv‐sh‐BACH2 and Lv‐sh‐FUS, constructed the lentiviral vector containing sh‐NC synchronously, obtaining the lentiviral Lv‐sh‐NC as a NC. The TSLNC8 sequence was recombined into the lentiviral vector pLenti6/V5‐DEST after amplification by PCR, using the pLenti6.3/V5eDEST Gateway Vector Kit (Thermo Fisher Scientific). After the identification was correct, HEK‐293T cells were transfected with the ViraPower packaging mixture. After 48h, the virus supernatant was collected, concentrated and tittered obtaining the recombinant lentivirus Lv‐TALNC8; lentivirus empty vector was packaged synchronously, and Lv‐EV was used as a NC. U87 and U251 cells were transfected with lentivirus obtained as described above, and cells stably expressing sh‐NC+EV, sh‐BACH2, sh‐FUS, TSLNC8‐OE and sh‐BACH2+sh‐FUS+TSLNC8‐OE were selected. The gene levels for lentiviral stable transfections are shown in Fig. S1L‐N.

Four‐week‐old athymic nude mice (BALB/c) were purchased from the Cancer Institute of the China Academy of Medical Science. The nude mice were divided into six groups (control, sh‐NC+EV, sh‐BACH2, sh‐FUS, TSLNC8‐OE and sh‐BACH2+sh‐FUS+TSLNC8‐OE) with eight mice in each group. To establish the subcutaneous xenograft glioma model, the nude mice were subcutaneously injected with 3 × 10^5^ cells into the right limb area to establish the subcutaneous glioma model. The change in the volume of the transplanted tumours was detected every 5 days according to the formula: volume (mm^3^) = length × width^2^/2. Subsequently, 45 days after injection, the mice were sacrificed by the intraperitoneal injection of an overdose of pentobarbital physiological saline (200 mg·kg^−1^). The tumours were dissected and separated in a laboratory operating room. To establish the orthotopic glioma model, nude mice were anaesthetised by intraperitoneal injection of 2% sodium pentobarbital (40 mg·kg^−1^), fixed on the brain stereotaxic instrument. After sterilising the skin on the top of the head with 75% ethanol, a vertical incision was made at the midcranial line after the eye fissure to expose the skull. The inoculation site was 3 mm beside midline of coronal suture, carefully drilled through the skull with an animal skull drill and then vertically entered to the brain 4.5 mm through the hole. With the use of microinjector, 3 × 10^5^ cells (5 µL) were injected into the right caudate nucleus of the nude mouse brain, followed by then dynamic observation of survival status, survival time and survival curves were drawn.

### 2.16 Statistical analysis

All data are presented as the mean ± SD and statistical from three independent experiments. Results were statistically analysed using Student's *t*‐test or one‐way ANOVA in spss 19.0 software (IBM, Armonk, NY, USA). *P* < 0.05 was considered statistically different.

## 3 Results

### 3.1 BACH2 and FUS interaction promotes the malignant biological behaviour of glioma cells

**Fig. 1:**
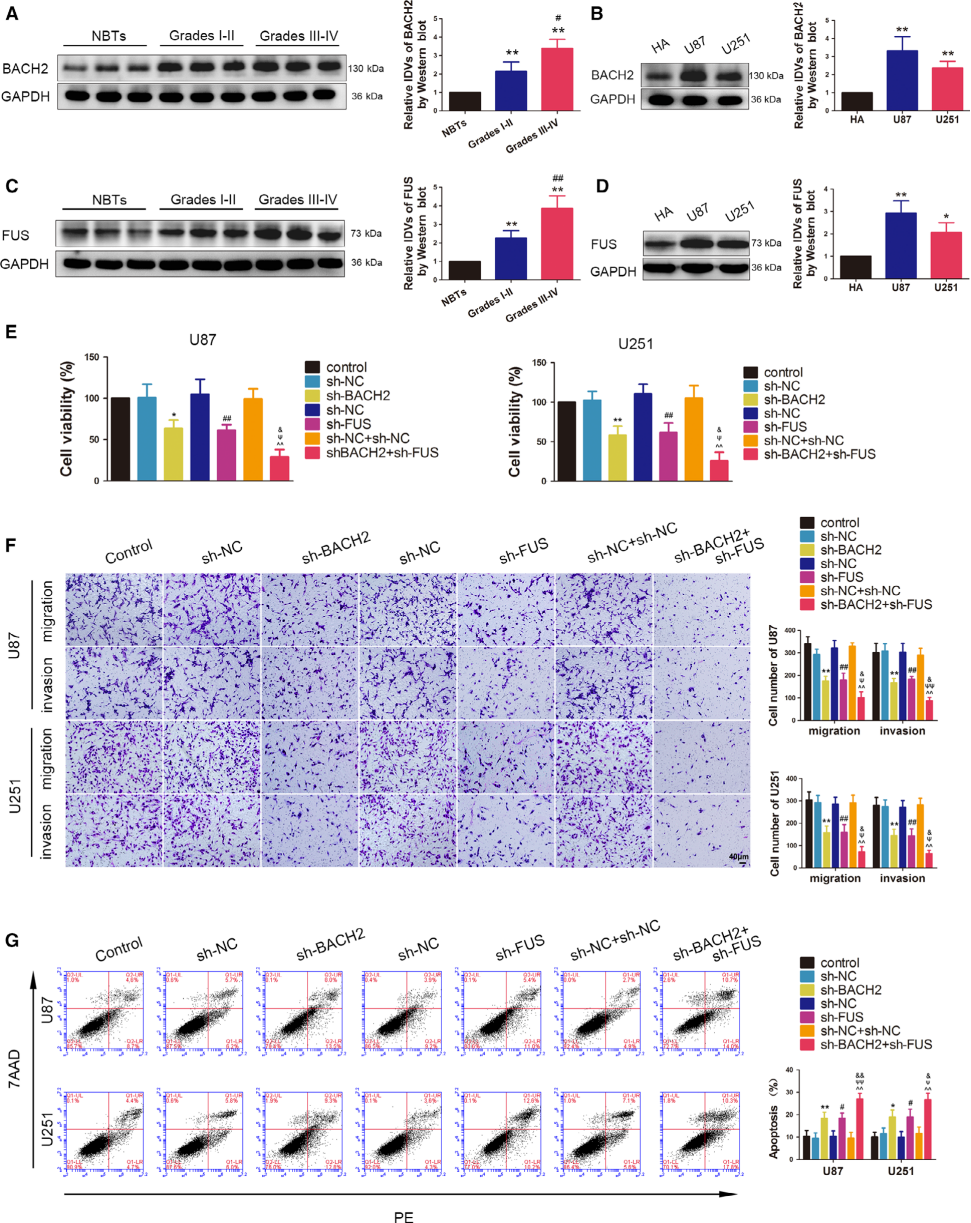
The endogenous expression of BACH2 and FUS, and their effects on the biological behaviour of glioma cells. (A) The expression of BCACH2 was measured by western blotting in NBTs and glioma tissues of grade Ⅰ–Ⅱ and grade Ⅲ–Ⅳ. Data are presented as mean ± SD (*n* = 12 for each group) and analysed by using one‐way ANOVA, **P < 0.01 vs. NBT group; ^#^ *P* < 0.05 vs. Grade Ⅰ‐Ⅱ group. (B) The expression of BACH2 was measured by western blotting in normal HA and glioblastoma cell lines (U87 and U251). Data are presented as mean ± SD (*n* = 3 for each group) and analysed by using one‐way ANOVA. **P < 0.01 vs. HA. (C) The expression of FUS was measured by western blotting in NBTs and glioma tissues of grade Ⅰ–Ⅱ and grade Ⅲ–Ⅳ. Data are presented as mean ± SD (*n* = 12 for each group) and analysed by using one‐way ANOVA. **P < 0.01 vs. NBT group; ^##^ *P* < 0.01 vs. grade Ⅰ‐Ⅱ group. (D) The expression of FUS was measured by western blotting in normal HA, U87 and U251 cells. Data are presented as mean ± SD (*n* = 3 for each group) and analysed by using one‐way ANOVA. *P < 0.05 vs. HA; **P < 0.01 vs. HA. (E) CCK‐8 assay was used to measure the effect of BACH2 and FUS on the viability of U87 and U251 cells. (F) Transwell assays were used to measure the effect of BACH2 and FUS on cell migration and invasion of U87 and U251 cells. (G) Flow cytometry analysis of U87 and U251 cells treated with altered expressions of BACH2 and FUS. (E‐G) Data are presented as mean ± SD (*n* = 3 for each group) and analysed by using one‐way ANOVA. *P < 0.05 vs. sh‐NC group; **P < 0.01 vs. sh‐NC group; ^#^ *P* < 0.05 vs. sh‐NC group; ^##^ *P* < 0.01 vs. sh‐NC group; ^^^^ *P* < 0.01 vs. sh‐NC+sh‐NC group; ^ψ^ *P* < 0.05 vs. sh‐BACH2 group; ^ψψ^ *P* < 0.01 vs. sh‐BACH2 group; ^&^ *P* < 0.05 vs. sh‐FUS group; ^&&^ *P* < 0.01 vs. sh‐FUS group. Scale bar represents 40 μm.

According to the data (GSE7696, GSE4290) obtained from the GEO database, the expression of BACH2 and FUS was both elevated in gliomas compared with the normal tissues (Fig. S2A‐B). The protein expression of BACH2 and FUS in different grades of glioma tissues and HA cells and U87 and U251 glioma cells was detected by western blotting (Fig. 1A–D). As shown in Fig. 1A,C, compared with NBTs, the expression of BACH2 and FUS was significantly increased in glioma tissues and with increasing pathological grade. Moreover, compared with HA cells, a significant upregulation of BACH2 and FUS protein expression was found in both U87 and U251 glioma cells (Fig. 1B,D).

To further clarify the effects of BACH2 and FUS on the malignant biological behaviour of glioma cells, stable BACH2 or FUS knockdown and both BACH2 and FUS knockdown U87 and U251 cells were constructed. Three shRNAs against BACH2 or FUS different sequence sites were transiently transfected. Site #2 of BACH2 shRNAs and site #1 of FUS shRNAs showed the highest knockdown efficiency in qRT‐PCR after 48 h of transient transfection and were selected to perform stable transfections in subsequent experiments (Fig. S1A–D). The results of the cell counting kit‐8 (CCK‐8) assay showed that BACH2 or FUS knockdown led to decreased cell viability in U87 and U251 cells. The migration and invasion abilities of U87 and U251 cells were also decreased, while the apoptosis rate was increased after BACH2 or FUS knockdown. Furthermore, compared with the sh‐BACH2 or sh‐FUS groups, the effects were enhanced in the sh‐BACH2+sh‐FUS group (Fig. 1E–G).

**Fig. 2:**
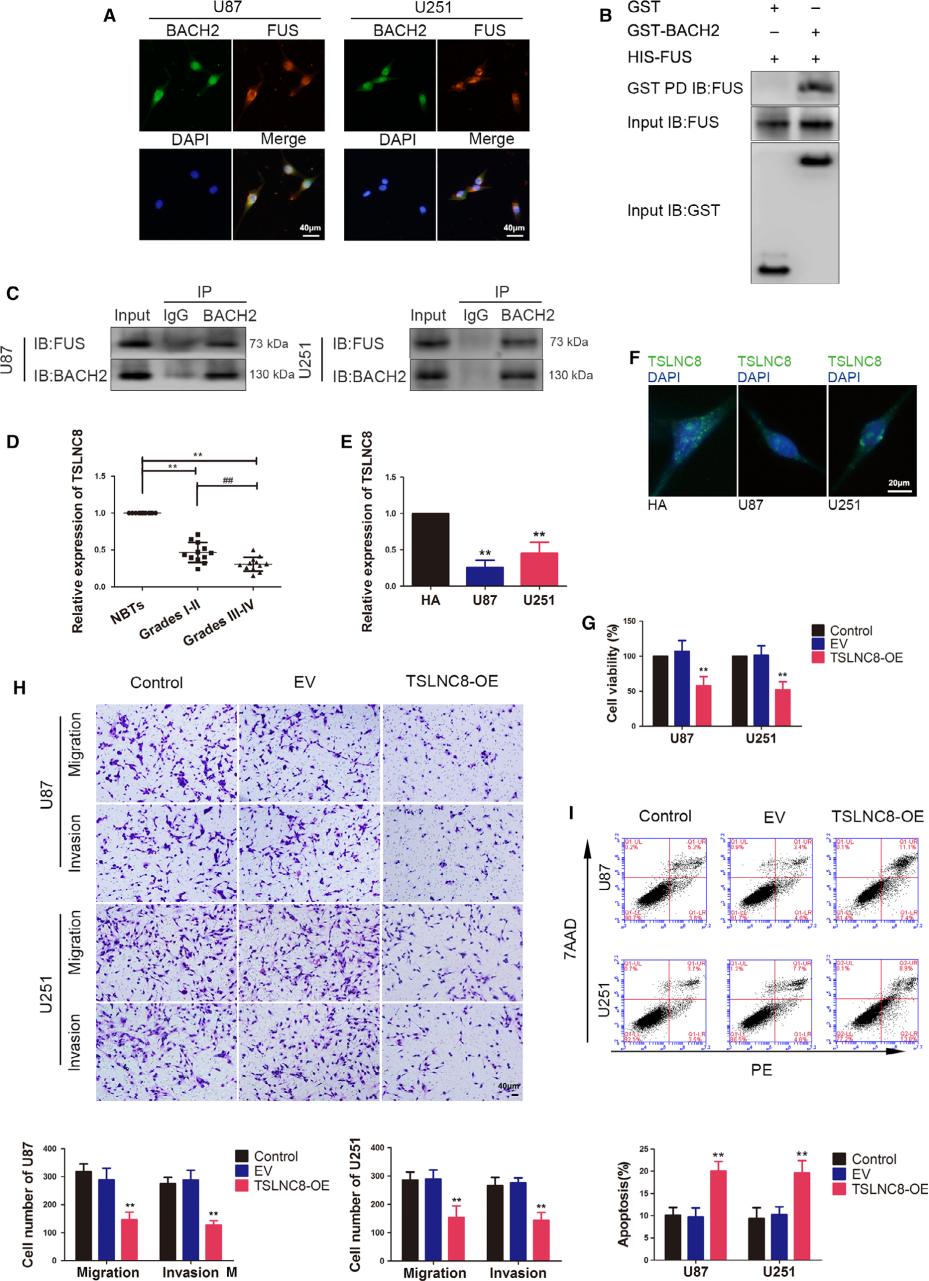
Colocalisation and interaction between BACH2 and FUS, the endogenous expression of TSLNC8 and its effects on the biological behaviour of glioma cells. (A) IF microscopy images of the cellular colocalisation of BACH2 and FUS proteins in U87 and U251 cells (*n* = 1). Scale bar represents 40 μm. (B) GST pulldown was used to determine the interaction between BACH2 and FUS *in vitro* (*n* = 1). (C) Glioma cells were subjected to immunoprecipitation using anti‐IgG or anti‐BACH2, followed by immunoblotting with anti‐FUS and anti‐BACH2 (*n* = 1). (D) qRT–PCR was used to measure the expression of TSLNC8 in NBTs and glioma tissues of grade Ⅰ–Ⅱ and grade Ⅲ–Ⅳ. Data are presented as mean ± SD (*n* = 12 for each group) and analysed by using one‐way ANOVA. **P < 0.01 vs. NBT group; ^##^ *P* < 0.01 vs. grade Ⅰ–Ⅱ group. (E) Expression levels of TSLNC8 in normal HA, U87 and U251 cells. Data are presented as mean ± SD (*n* = 3 for each group) and analysed by using one‐way ANOVA. **P < 0.01 vs. HA group. (F) FISH microscopy images of the cellular distribution of TSLNC8 in normal HA, U87 and U251 cells (*n* = 1). Scale bar represents 20 μm. (G) CCK‐8 assay was used to measure the effect of TSLNC8 on the viability of U87 and U251 cells. (H) Transwell assays were used to measure the effect of TSLNC8 on cell migration and invasion of U87 and U251 cells. (I) Flow cytometry analysis of U87 and U251 cells treated with altered expression of TSLNC8. (G–I) Data are presented as mean ± SD (*n* = 3 for each group) and analysed by using one‐way ANOVA. **P < 0.01 vs. EV group. Scale bar represents 40 μm.

Based on these results, an immunofluorescence assay, GST pull‐down assay and Co‐IP assay were performed to determine whether BACH2 and FUS interacted. An immunofluorescence assay was used to detect the localisation of BACH2 and FUS in glioma cells. As shown in Fig. 2A, BACH2 with green fluorescence and FUS with red fluorescence were localised in both the cytoplasm and nucleus, although mainly in the nucleus. Furthermore, the colocalisation of BACH2 and FUS was observed in the merged images. Next, a GST pull‐down assay was performed, and detected a combination in the GST‐BACH2+His‐FUS group, but not in the NC GST+His‐FUS group (Fig. 2B). The binding *in vivo* was further verified by Co‐IP assay (Fig. 2C).

### 3.2 Overexpression of TSLNC8 inhibits the malignant biological behaviour of glioma cells and BACH2 transcriptionally suppresses TSLNC8 expression in glioma cells

The expression profile from lncRNA analysis of U87 and U251 cells upon BACH2 knockdown revealed the upregulation of several lncRNAs (Fig. S2C), and lncRNAs with a more than twofold change were validated by qRT‐PCR. Interestingly, TSLNC8 showed a three to fourfold change (Fig. S2D). The expression of TSLNC8 in different glioma tissues, HA cells, and U87 and U251 glioma cells was detected by qRT‐PCR. Compared with NBTs, TSLNC8 expression was significantly decreased in glioma tissues, and the expression levels were progressively reduced with increasing grade. In addition, TSLNC8 expression was reduced in U87 and U251 cells compared with that in HA cells (Fig. 2D,E). Furthermore, fluorescence *in situ* hybridisation (FISH) showed that TSLNC8 was observed in both the cytoplasm and nucleus (Fig. 2F).

To analyse the potential effects of TSLNC8 on the malignant biological behaviour of glioma cells, TSLNC8 was overexpressed in U87 and U251 cells (gene expression level for transfection is shown in Fig. S1E). The results showed that the viability, migration and invasion abilities of U87 and U251 cells were significantly decreased in the TSLNC8‐OE (overexpression) group (Fig. 2G,H), and the apoptosis rate was significantly increased compared with the EV (empty vector) group (Fig. 2I). Interestingly, TSLNC8 knockdown had the opposite effect on the malignant biological behaviour of glioma cells (Fig. S2H–J).

**Fig. 3:**
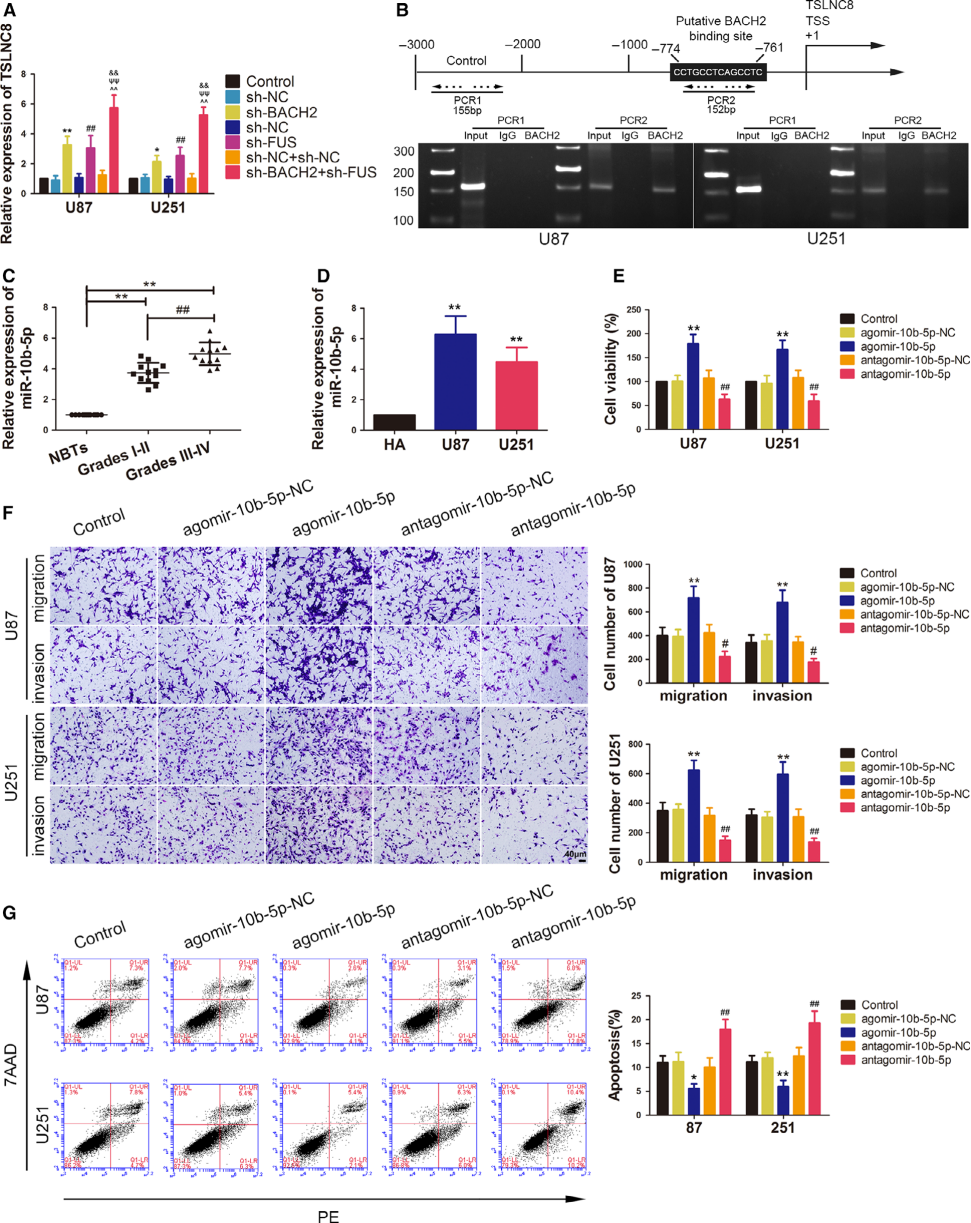
Endogenous expression of miR‐10b‐5p and its effects on the biological behaviour of glioma cells. (A) qRT–PCR was used to measure the expression of TSLNC8 in U87 and U251 cells, which were treated by knockdown of BACH2 and FUS. Data are presented as mean ± SD (*n* = 3 for each group) and analysed by using one‐way ANOVA. *P < 0.05 vs. sh‐NC group; **P < 0.01 vs. sh‐NC group; ^##^ *P* < 0.01 vs. sh‐NC group; ^^^^ *P* < 0.01 vs. sh‐NC+sh‐NC group; ^ψψ^ *P* < 0.01 vs. sh‐BACH2 group; ^&&^ *P* < 0.01 vs. sh‐FUS group. (B) BACH2 binds to the promoter of TSLNC8 in U87 and U251 cells. A schematic representation of the human BACH2 promoter region 3000‐bp upstream of the TSS, which was designated as +1. Putative BACH2‐binding sites are illustrated. Immunoprecipitated DNA was amplified by PCR. Normal rabbit IgG was used as a NC (*n* = 1). (C) qRT–PCR was used to measure the expression of miR‐10b‐5p in NBTs and glioma tissues of grade I–II and grade III–IV. Data are presented as mean ± SD (*n* = 12 for each group) and analysed by using one‐way ANOVA. **P < 0.01 vs. NBT group; ^##^ *P* < 0.01 vs. grade Ⅰ–Ⅱ group. (D) Expression levels of miR‐10b‐5p in normal HA, U87 and U251 cells. Data are presented as mean ± SD (*n* = 3 for each group) and analysed by using one‐way ANOVA. **P < 0.01 vs. HA group. (E) The CCK‐8 assay was used to measure the effect of miR‐10b‐5p on the proliferation of U87 and U251 cells. (F) Transwell assays were used to measure the effect of miR‐10b‐5p on the migration and invasion of U87 and U251 cells. (G) Flow cytometry analysis of U87 and U251 cells treated with altered expression of miR‐10b‐5p. (E–G) Data are presented as mean ± SD (*n* = 3 for each group) and analysed by using one‐way ANOVA. *P < 0.05 vs. agomir‐10b‐5p‐NC group; **P < 0.01 vs. agomir‐10b‐5p‐NC group; ^#^ *P* < 0.05 vs. antagomir‐10b‐5p‐NC group, ^##^ *P* < 0.01 vs. antagomir‐10b‐5p‐NC group. Scale bar represents 40 μm.

Importantly, TSLNC8 expression was detected after BACH2 or FUS knockdown alone or in both knockdowns. Compared with the sh‐NC and sh‐NC+sh‐NC groups, TSLNC8 expression in the sh‐BACH2, sh‐FUS or sh‐BACH2+sh‐FUS groups was found to be significantly increased. Moreover, compared with the sh‐BACH2 or sh‐FUS groups, TSLNC8 expression was also increased in the sh‐BACH2+sh‐FUS group (Fig. 3A), suggesting that FUS knockdown enhanced the inhibitory effect of BACH2 knockdown.

A putative binding site for BACH2 in the promoter region of TSLNC8 was predicted using JASPAR, a bioinformatic database. A chromatin immunoprecipitation (ChIP) assay was performed to validate this hypothesis. As shown in Fig. 3B, BACH2 was found to bind to the putative binding site in the −774‐ to −761‐bp region of the TSLNC8 promoter, compared with the NC without putative binding sites in the −3000 to −2000 bp upstream of the transcription start site (TSS) of TSLNC8.

### 3.3 miR‐10b‐5p is upregulated in glioma tissues and cells, while miR‐10b‐5p knockdown inhibits the malignant biological behaviour of glioma cells

The miRNA microarray revealed that miR‐10b‐5p was significantly downregulated in glioma cells treated with TSLNC8‐OE. qRT‐PCR was performed to validate several downregulated miRNAs in U87 and U251 glioma cells (Fig. S2E,F). The expression of miR‐10b‐5p in the glioma tissue and cells was further detected using qRT‐PCR. The results showed that miR‐10b‐5p expression was significantly increased in glioma tissues and U87 and U251 glioma cells, while the expression levels were progressively higher with increasing grade in glioma tissues (Fig. 3C,D).

To determine the influence of miR‐10b‐5p on the malignant biological behaviour of glioma cells, miR‐10b‐5p overexpression or the inhibition of transient transfection in U87 and U251 cells was carried out (gene expression levels for transfection are shown in Fig. S1F,G). As shown in Fig. 3E–G, compared with the agomir‐10b‐5p‐NC group, the cell viability, migration and invasion abilities of the glioma cells were significantly increased in the agomir‐10b‐5p group, while the apoptosis rate was significantly decreased. In contrast, compared with the antagomir‐10b‐5p‐NC group, the antagomir‐10b‐5p group induced a significant decrease in cell viability, migration and invasion in U87 and U251 glioma cells, but an increase in cell apoptosis.

### 3.4 miR‐10b‐5p may participate in the inhibition induced by TSLNC8 overexpression on the malignant biological behaviour of glioma cells

**Fig. 4:**
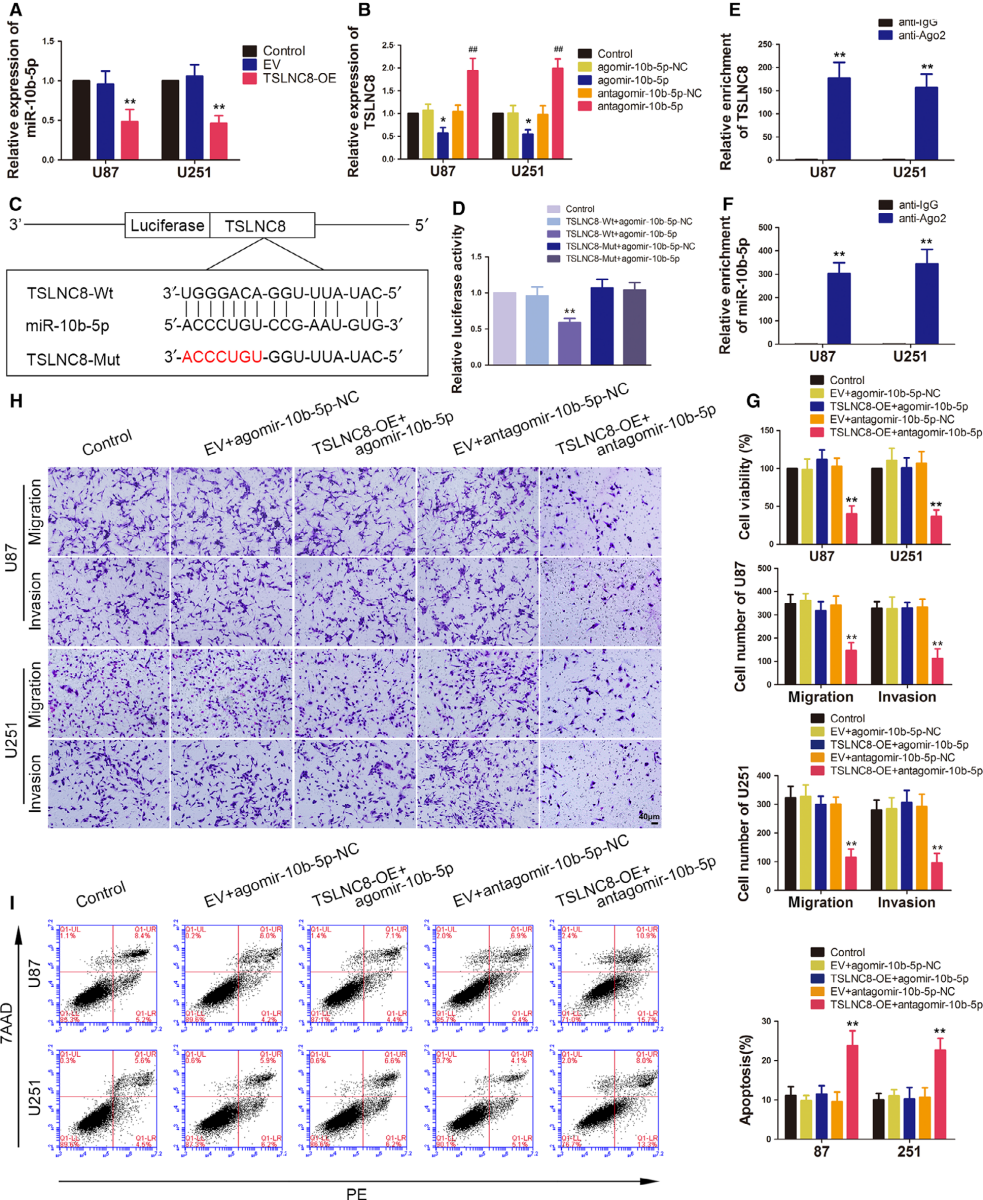
Overexpression of TSLNC8 impaired malignant biological behaviours of glioma cells by reducing miR‐10b‐5p expression. (A) qRT–PCR analysis of miR‐10b‐5p expression, which is regulated by TSLNC8 in U87 and U251 cells. Data are presented as mean ± SD (*n* = 3 for each group) and analysed by using one‐way ANOVA. **P < 0.01 vs. EV group. (B) qRT–PCR analysis of TSLNC8 expression regulated by overexpression or knockdown of miR‐10b‐5p in U87 and U251 cells. Data are presented as mean ± SD (*n* = 3 for each group) and analysed by using one‐way ANOVA. *P < 0.05 vs. agomir‐10b‐5p‐NC group; ^##^ *P* < 0.01 vs. antagomir‐10b‐5p‐NC group. (C) The predicted binding sites of miR‐10b‐5p in TSLNC8‐Wt and the designed mutant sequence of TSLNC8‐Mut are indicated. (D) Relative luciferase activity of TSLNC8‐Wt or TSLNC8‐Mut and agomir‐10b‐5p‐NC or agomir‐10b‐5p cotransfected HEK293 cells. Data are presented as mean ± SD (*n* = 3 for each group) and analysed by using one‐way ANOVA. **P < 0.01 vs. TSLNC8‐Wt+agomir‐10b‐5p‐NC group. (E, F) RIP confirmed that TSLNC8 and miR‐10b‐5p were in the RISC complex. Data are presented as mean ± SD (*n* = 3 for each group) and analysed by using Student's *t*‐test. **P < 0.01 vs. anti‐IgG group. (G) CCK‐8 assay was used to measure the effect of TSLNC8 and miR‐10b‐5p on the viability of U87 and U251 cells. (H) Transwell assays were used to measure the effect of TSLNC8 and miR‐10b‐5p on the migration and invasion of U87 and U251 cells. (I) Flow cytometry analysis of U87 and U251 cells treated with altered expression of TSLNC8 and miR‐10b‐5p. (G–I) Data are presented as mean ± SD (*n* = 3 for each group) and analysed by using one‐way ANOVA. **P < 0.01 vs. EV+antagomir‐10b‐5p ‐NC group. Scale bar represents 40 μm.

miR‐10b‐5p expression was detected following TSLNC8 overexpression and was found to be decreased in the TSLNC8‐OE group (Fig. 4A). However, while the expression of TSLNC8 in the agomir‐10b‐5p group was significantly reduced, it was increased in the antagomir‐10b‐5p group (Fig. 4B).

A putative binding site between TSLNC8 and miR10b‐5p was predicted using the bioinformatic database DIANA (Fig. 4C) and confirmed using a dual‐luciferase reporter system assay. The results showed that the relative luciferase activity of the TSLNC8‐Wt+agomir‐10b‐5p group was significantly lower than that of the TSLNC8‐Wt+agomir‐10b‐5p‐NC group, while the relative luciferase activity was not significantly different between the TSLNC8‐Mut+agomir‐10b‐5p and TSLNC8‐Mut+agomir‐10b‐5p‐NC groups (Fig. 4D). To clarify whether TSLNC8 and miR‐10b‐5p co‐existed in RNA‐induced silencing complex (RISC), we performed a RIP assay. As shown in Fig. 4E‐F, the expression levels of TSLNC8 and miR‐10b‐5p were both significantly increased in the anti‐argonaute‐2 group.

The effects of TSLNC8 and miR‐10b‐5p on the biological behaviour of glioma cells showed that the TSLNC8‐OE+antagomir‐10b‐5p group resulted in a decrease in cell viability, migration and invasion, but an increase in apoptotic rate compared with the EV+antagomir‐10b‐5p‐NC group. There were no significant differences between the TSLNC8 ‐OE+agomir‐10b‐5p and EV+agomir‐10b‐5p‐NC groups (Fig. 4G‐I). These results indicated that the overexpression of TSLNC8 alone and in combination with the overexpression of miR‐10b‐5p reversed the inhibitory effect of TSLNC8 ‐OE+antagomir‐10b‐5p on glioma cell biological behaviour.

### 3.5 WWC3 inhibits the malignant biological behaviour of glioma cells by promoting YAP phosphorylation to reduce its quantity in the nucleus

**Fig. 5:**
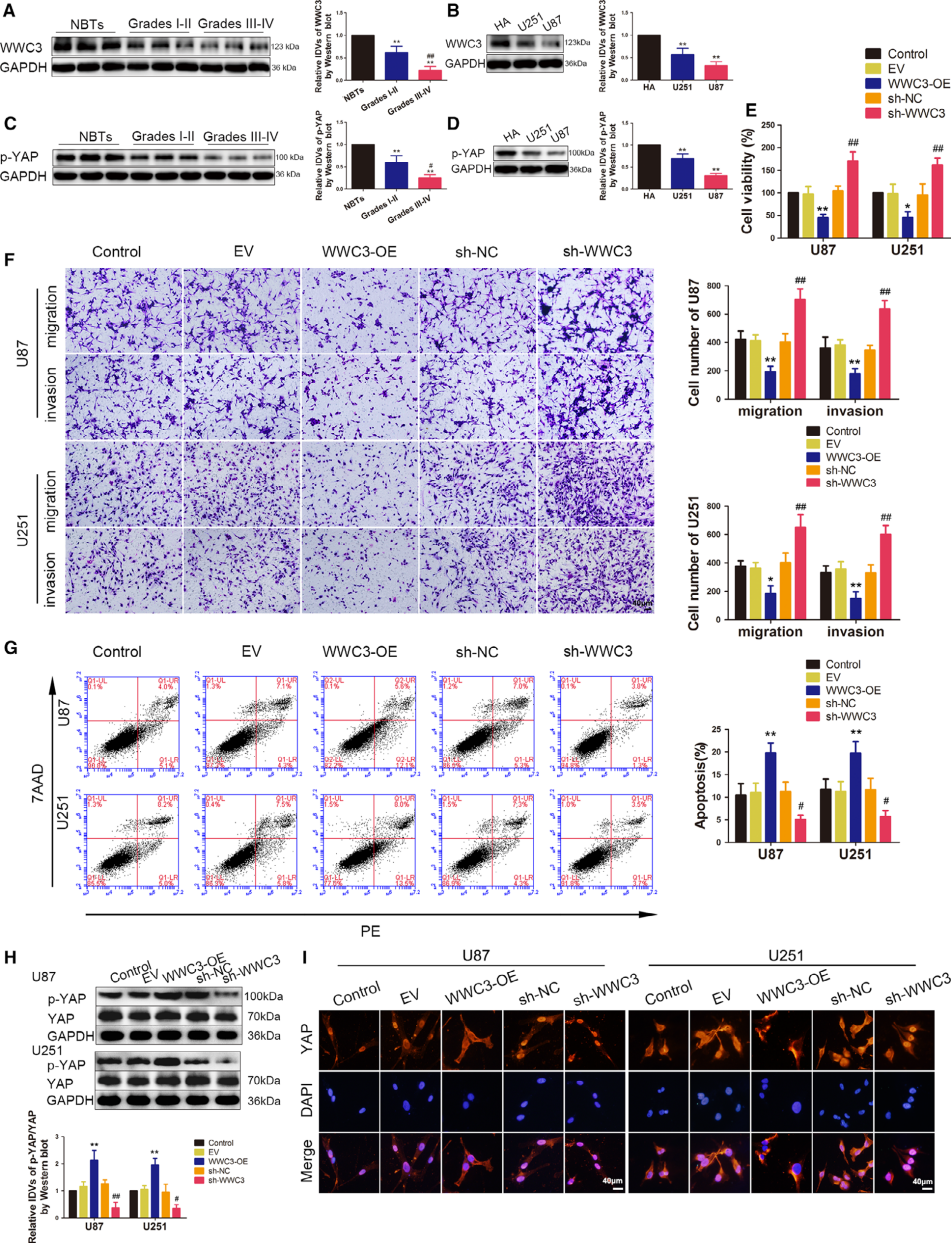
Endogenous expression of WWC3 and the effects of WWC3 on the biological behaviour of glioma cells. (A) The expression of WWC3 was measured by western blotting in NBT and glioma tissues of grade Ⅰ–Ⅱ and grade Ⅲ–Ⅳ. Data are presented as mean ± SD (*n* = 9, each group) and analysed by using one‐way ANOVA. **P < 0.01 vs. NBT group; ^##^ *P* < 0.01 vs. grade I‐II group. (B) The expression of WWC3 was measured by western blotting in normal HA, U87 and U251 cells. Data are presented as mean ± SD (*n* = 3 for each group) and analysed by using one‐way ANOVA. **P < 0.01 vs. HA. (C) The p‐YAP level was measured by western blot in NBTs and glioma tissues of grade I‐II and grade III‐IV. Data are presented as mean ± SD (*n* = 9 for each group) and analysed by using one‐way ANOVA. **P < 0.01 vs. NBTs; ^#^ *P* < 0.05 vs. grade I‐II group. (D) The p‐YAP level was measured by Western blot in normal HA, U87 and U251 cells. Data are presented as mean ± SD (*n* = 3 for each group) and analysed by using one‐way ANOVA. **P < 0.01 vs. HA group. (E) CCK‐8 assay was used to measure the effect of WWC3 on the proliferation of U87 and U251 cells. (F) Transwell assays were used to measure the effect of WWC3 on cell migration and invasion of U87 and U251 cells. (G) Flow cytometry analysis of U87 and U251 cells treated with altered expression of WWC3. (E–G) Data are presented as mean ± SD (*n* = 3 for each group) and analysed by using one‐way ANOVA. *P < 0.05 vs. EV group; **P < 0.01 vs. EV group; ^#^ *P* < 0.05 vs. sh‐NC group; ^##^ *P* < 0.01 vs. sh‐NC group. Scale bar represents 40 μm. (H) Western blotting assay was used to measure the p‐YAP level in U87 and U251 cells, which treated by WWC3 overexpression or knockdown. Data are presented as mean ± SD (*n* = 3 for each group) and analysed by using one‐way ANOVA. **P < 0.01 vs. EV group; ^#^ *P* < 0.05 vs. sh‐NC group; ^##^ *P* < 0.01 vs. sh‐NC group. (I) IF microscopy images of the YAP distribution in the nucleus and cytoplasm, which treated by WWC3 overexpression or knockdown (*n* = 1). Scale bars represent 40 μm.

Recent studies have demonstrated that WWC3 regulates the Wnt and Hippo pathways via Dvl protein and LATS1 to inhibit the invasion and metastasis of lung cancer cells [23]. The expression of WWC3 is downregulated in human glioma tissues and cells [24]. Western blotting was used to validate the expression of WWC3. The results showed that the protein levels of WWC3 were low in different glioma tissues, progressively reduced with increasing grade and downregulated in U87 and U251 glioma cells (Fig. 5A,B). Considering that YAP is a crucial downstream protein of the Hippo pathway, the YAP phosphorylation (p‐YAP) level was determined in glioma tissues and cells. As shown in Fig. 5C,D, the p‐YAP level was significantly decreased in glioma tissues of different grades and decreased more in grade III‐IV glioma tissues than in grade I‐II glioma tissues. Consistently, the p‐YAP level was decreased in U87 and U251 glioma cells.

Furthermore, the function of WWC3 on the malignant biological behaviour of glioma cells was assessed. To this end, U87 and U251 glioma cells were treated with WWC3‐OE and sh‐WWC3 plasmids and their corresponding negative plasmids (gene expression levels for transfection, which were detected by qPCR and western blotting, are shown in Fig. S1H–K). As shown in Fig. 5E–G, WWC3 overexpression was found to exert a significant inhibitory effect on the cell viability, migration and invasion of U87 and U251 glioma cells, but promoted cell apoptosis. The WWC3 knockdown promoted the cell viability, migration and invasion of U87 and U251 glioma cells, but suppressed cell apoptosis.

Subsequently, the p‐YAP levels in the U87 and U251 glioma cells were detected using western blotting after the overexpression or knockdown of WWC3. Compared to the EV group, the p‐YAP levels were significantly increased in the WWC3‐OE group. Compared to sh‐NC, the p‐YAP levels were decreased in the sh‐WWC3 group, whereas the total YAP protein levels did not change significantly (Fig. 5H). Since p‐YAP may affect the nucleoplasmic distribution of YAP, the differential distribution of total YAP in the nucleus and cytoplasm was detected. Consistent with the p‐YAP levels, the expression of YAP in the cytoplasm increased with WWC3 overexpression, but decreased in the nucleus, while the knockdown of WWC3 expression produced the opposite effect (Fig. S2K,L). The results of the immunofluorescence assay further validated the effects above, wherein the enrichment of YAP was found to increase in the cytoplasm and decrease in the nucleus after WWC3 overexpression. YAP was more highly expressed in the nucleus than in the cytoplasm after WWC3 knockdown (Fig. 5I).

### 3.6 miR‐10b‐5p downregulates WWC3 expression by binding to WWC3 3′‐UTR，WWC3 attenuated the promotion induced by miR‐10b‐5p overexpression on the malignant biological behaviour of glioma cells

**Fig. 6:**
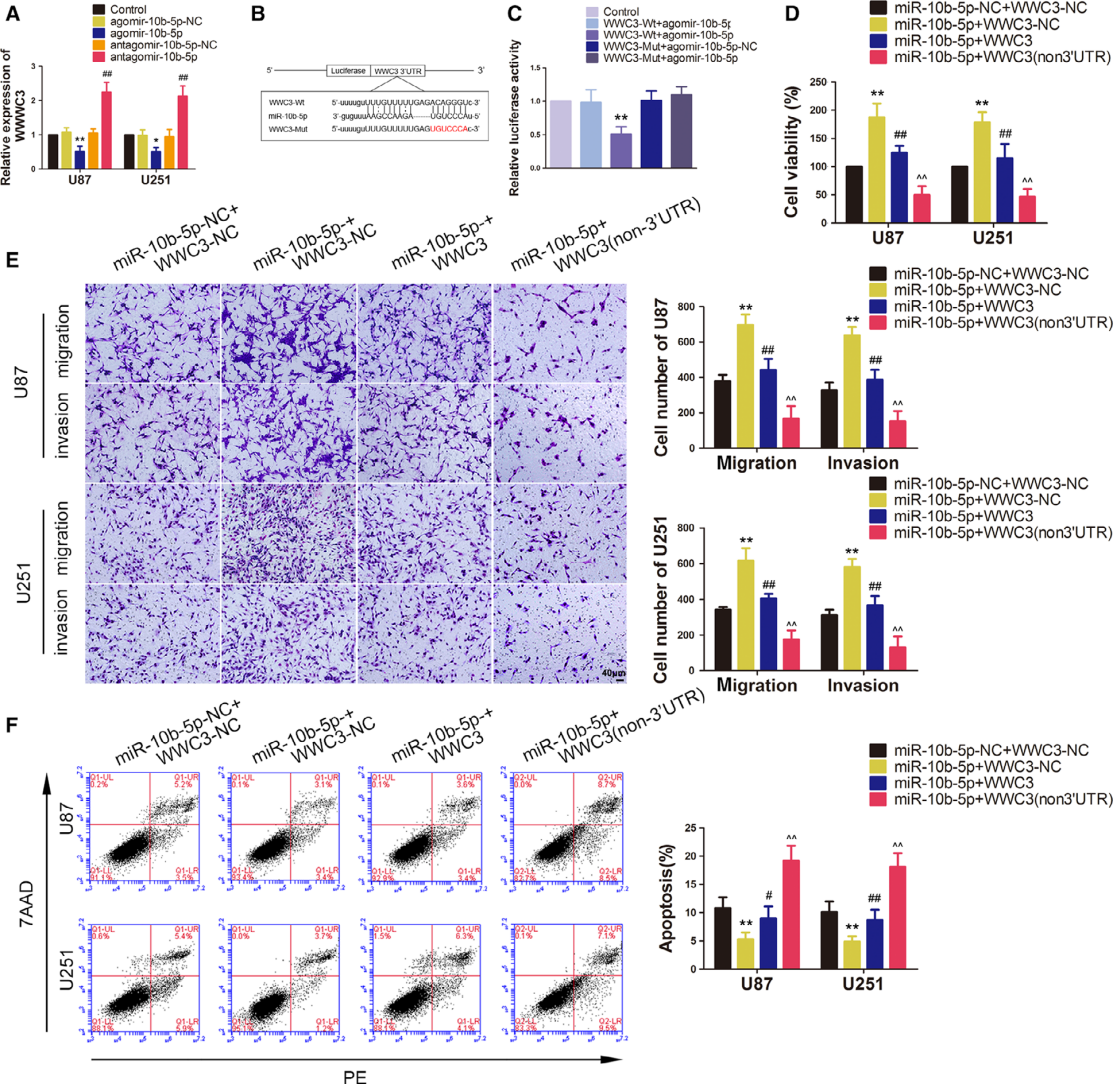
miR‐10b‐5p plays an oncogenic role in glioma cells by binding to the WWC3 3′‐UTR. (A) qRT–PCR analysis of WWC3 expression, which is regulated by miR‐10b‐5p in U87 and U251 cells. Data are presented as mean ± SD (*n* = 3 for each group) and analysed by using one‐way ANOVA. *P < 0.05 vs. agomir‐10b‐5p‐NC group; **P < 0.01 vs. agomir‐10b‐5p‐NC group; ^##^ *P* < 0.01 vs. antagomir‐10b‐5p‐NC group. (B) The predicted binding sites of miR‐10b‐5p in the 3′‐UTR region of WWC3‐Wt and the designed mutant sequence of WWC3‐Mut are indicated. (C) Relative luciferase activity of WWC3‐Wt or WWC3‐Mut and agomir‐10b‐5p‐NC or agomir‐10b‐5p cotransfected HEK293 cells. Data are presented as mean ± SD (*n* = 3 for each group) and analysed by using one‐way ANOVA. **P < 0.01 vs. WWC3‐Wt+agomir‐10b‐5p‐NC group. (D) CCK‐8 assay was used to measure the effect of miR‐10b‐5p and WWC3 on the viability of U87 and U251 cells. (E) Transwell assays were used to measure the effect of miR‐10b‐5p and WWC3 on cell migration and invasion of U87 and U251 cells. (F) Flow cytometry analysis of the effect of miR‐10b‐5p and WWC3 on apoptosis in U87 and U251 cells. (D‐F) Data are presented as mean ± SD (*n* = 3 for each group) and analysed by using one‐way ANOVA. **P < 0.01 vs. miR‐10b‐5p‐NC+WWC3‐NC group; ^#^ *P* < 0.05, ^##^ *P* < 0.01 vs. miR‐10b‐5p+WWC3‐NC group; ^^^^ *P* < 0.01 vs. miR‐10b‐5p+WWC3 group. Scale bar represents 40 μm.

The expression of WWC3 mRNA was prominently reduced following miR‐10b‐5p overexpression and was increased following miR‐10b‐5p knockdown (Fig. 6A). According to the bioinformatic database Miranda, the 3′‐UTR of WWC3 possessed a mir‐10b‐5p‐binding site (Fig. 6B). A dual‐luciferase reporter gene assay was performed to confirm this binding site. Compared with the WWC3‐Wt+agomir‐10b‐5p‐NC group, the relative luciferase activity was significantly decreased in the WWC3‐Wt+agomir‐10b‐5p group, while there was no statistically significant difference between the WWC3‐Mut+agomir‐10b‐5p and WWC3‐Mut+agomir‐10b‐5p‐NC groups (Fig. 6C).

To further validate the function of miR‐10b‐5p on WWC3, we assessed the effect of miR‐10b‐5p and WWC3 on the malignant biological behaviour of glioma cells. U87 and U251 glioma cells that overexpressed miR‐10b‐5p, WWC3 or WWC3 (non‐3′‐UTR) were constructed. Compared with miR‐10b‐5p‐NC+WWC3‐NC, the cell viability, migration and invasion abilities of U87 and U251 glioma cells were found to be significantly enhanced in the miR‐10b‐5p+WWC3‐NC group, but with attenuated cell apoptosis. Compared with the miR‐10b‐5p+WWC3‐NC group, the results of miR‐10b‐5p+WWC3 group showed that WWC3 reversed the promotion mediated by miR‐10b‐5p overexpression on glioma cell malignant biological behaviour. Moreover, compared with the miR‐10b‐5p+WWC3 group, the results of the miR‐10b‐5p+WWC3 (non‐3′‐UTR) group showed that the inhibition of WWC3 (non‐3′‐UTR) on malignant biological behaviour was stronger than that of WWC3 (Fig. 6D‐F). These results indicated that WWC3 reversed the promotion induced by miR‐10b‐5p overexpression on malignant biological behaviour of glioma cells and that the inhibition of WWC3‐non‐3′‐UTR was stronger than WWC3.

### 3.7 BACH2, FUS and TSLNC8 regulated WWC3 expression and YAP phosphorylation level by miR‐10b‐5p to impact the malignant biological behaviour of glioma cells

**Fig. 7:**
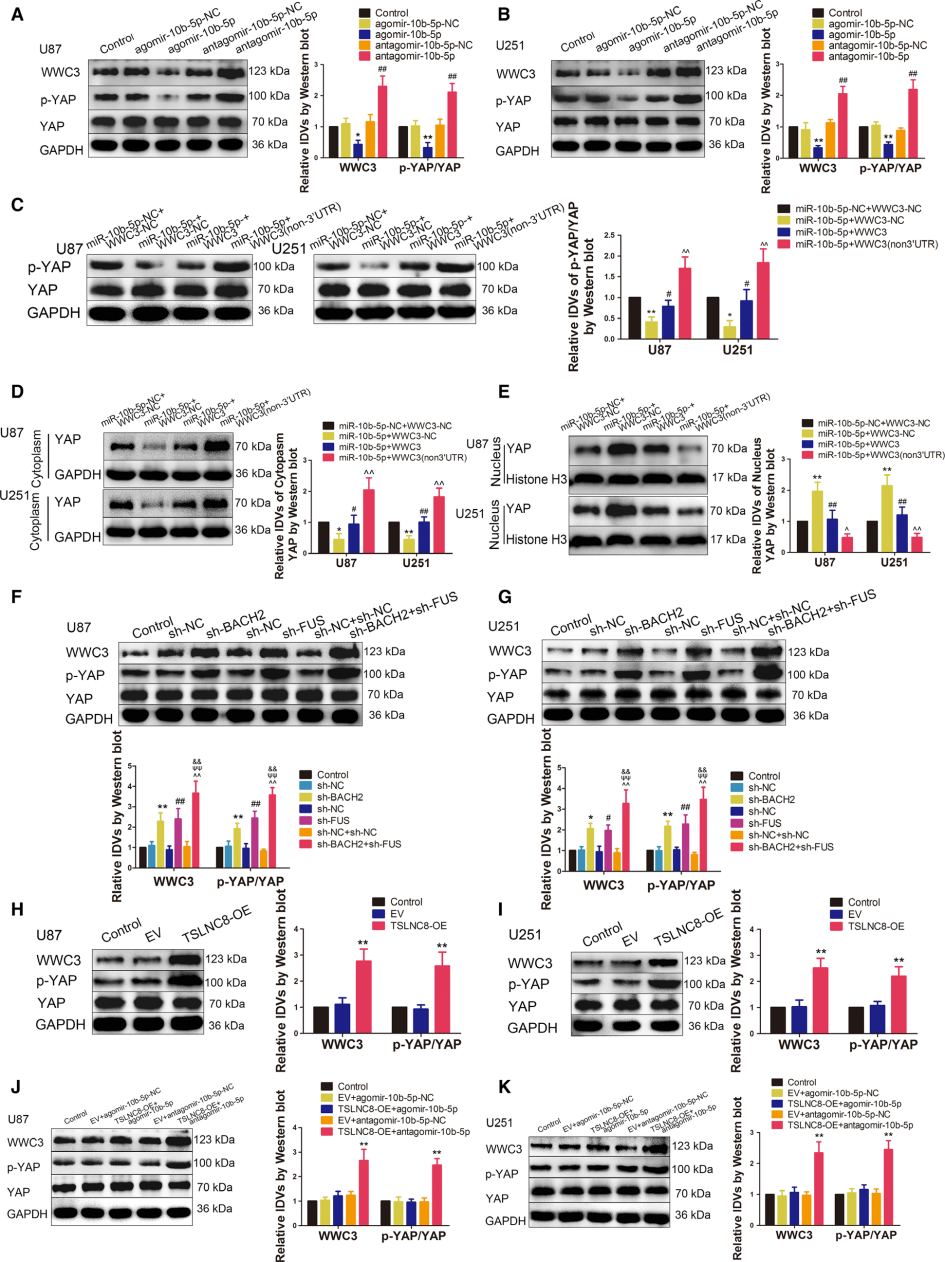
BACH2, FUS, TSLNC8 and miR‐10b‐5p regulated WWC3 expression and YAP phosphorylation levels. (A, B) Western blotting assay was used to measure WWC3 expression and p‐YAP levels in U87 and U251 cells treated with miR‐10b‐5p overexpression or knockdown. Data are presented as mean ± SD (*n* = 3 for each group) and analysed by using one‐way ANOVA. *P < 0.05, **P < 0.01 vs. agomir‐10b‐5p‐NC group; ^##^ *P* < 0.01 vs. antagomir‐10b‐5p‐NC group. (C) Western blotting assay was used to measure the p‐YAP level in U87 and U251 cells, which are regulated by miR10b‐5p targeting WWC3 3′‐UTR. Data are presented as mean ± SD (*n* = 3 for each group) and analysed by using one‐way ANOVA. *P < 0.05, **P < 0.01 vs. miR‐10b‐5p‐NC+WWC3‐NC group; ^#^ *P* < 0.05 vs. miR‐10b‐5p+WWC3‐NC group; ^^^^ *P* < 0.01 vs. miR‐10b‐5p+WWC3 group. (D) Western blotting assay was used to measure the cytoplasmic YAP expressions in U87 and U251 cells, which are regulated by miR10b‐5p targeting WWC3 3′‐UTR. Data are presented as mean ± SD (*n* = 3 for each group) and analysed by using one‐way ANOVA. *P < 0.05, **P < 0.01 vs. miR‐10b‐5p‐NC+WWC3‐NC group; ^#^ *P* < 0.05, ^##^ *P* < 0.01 vs. miR‐10b‐5p+WWC3‐NC group; ^^^^ *P* < 0.01 vs. miR‐10b‐5p+WWC3 group. (E) Western blotting assay was used to measure the nuclear YAP expressions in U87 and U251 cells, which are regulated by miR10b‐5p targeting WWC3 3′‐UTR. Data are presented as mean ± SD (*n* = 3 for each group) and analysed by using one‐way ANOVA. **P < 0.05 vs. miR‐10b‐5p‐NC+WWC3‐NC group; ^##^ *P* < 0.01 vs. miR‐10b‐5p+WWC3‐NC group; ^^^ *P* < 0.05, ^^^^ *P* < 0.01 vs. miR‐10b‐5p+WWC3 group. (F, G) Western blotting assay was used to measure WWC3 expressions and p‐YAP levels in U87 and U251 cells, which were treated by knockdown of BACH2 and FUS. Data are presented as mean ± SD (*n* = 3 for each group) and analysed by using one‐way ANOVA. *P < 0.05, **P < 0.01 vs. sh‐NC group; ^#^ *P* < 0.05, ^##^ *P* < 0.01 vs. sh‐NC group; ^^^^ *P* < 0.01 vs. sh‐NC+sh‐NC group; ^ψψ^ *P* < 0.01 vs. sh‐BACH2 group; ^&&^ *P* < 0.01 vs. sh‐FUS group. (H, I) Western blotting assay was used to measure WWC3 expressions and p‐YAP levels in U87 and U251 cells, which were treated with overexpressed TSLNC8. Data are presented as mean ± SD (*n* = 3 for each group) and analysed by using one‐way ANOVA. **P < 0.01 vs. EV group. (J, K) Western blotting assay was used to measure WWC3 expressions and p‐YAP levels regulated by TSLNC8 and miR10b‐5p in U87 and U251 cells. Data are presented as mean ± SD (*n* = 3 for each group) and analysed by using one‐way ANOVA. **P < 0.01 vs. EV+antagomir‐10b‐5p ‐NC group.

Western blotting was used to detect the effect of miR‐10b‐5p on WWC3, p‐YAP or YAP. As shown in Fig. 7A,B, the protein levels of WWC3 and p‐YAP levels were reduced after miR‐10b‐5p overexpression and elevated after miR‐10b‐5p knockdown. From the results of cotransfection miR‐10b‐5p overexpression with WWC3 or WWC3 (non‐3′‐UTR) overexpression (Fig. 7C), it was found that p‐YAP level was decreased by miR‐10b‐5p overexpression, and WWC3 could reverse the inhibition of YAP phosphorylation induced by miR‐10b‐5p overexpression alone, and WWC3 (non‐3′‐UTR) could increase more of YAP phosphorylation than WWC3, whereas the protein levels of total YAP were not changed significantly. In addition, the differential distribution of total YAP in the nucleus and cytoplasm was detected. In the miR‐10b‐5p+WWC3‐NC group, the expression of YAP in the cytoplasm was decreased, but increased in the nucleus, and WWC3 reversed this distribution. The reversed ability of WWC3 (non‐3′‐UTR) was stronger than WWC3 (Fig. 7D,E).

The knockdown of BACH2 and FUS alone increased the protein levels of WWC3 and p‐YAP in U87 and U251 cells, an effect which was magnified in the sh‐BACH2+sh‐FUS group (Fig. 7F,G). TSLNC8 overexpression was found to upregulate the protein levels of WWC3 and YAP phosphorylation (Fig. 7H,I). As shown in Fig. 7J,K, compared with the EV+antagomir‐10b‐5p‐NC group, the expression of WWC3 and the phosphorylation level of YAP in the TSLNC8‐OE+antagomir‐10b‐5p group were significantly increased, while there were no statistical differences between the TSLNC8‐OE+agomir‐10b‐5p and EV+agomir‐10b‐5p‐NC groups.

Taken together, these results indicate that BACH2 and FUS interaction enhanced the inhibition of the protein levels of WWC3 and YAP phosphorylation, TSLNC8 promoted the protein levels of WWC3 and YAP phosphorylation via miR‐10b‐5p and affected the malignant biological behaviour of glioma cells.

### 3.8 Knockdown of BACH2 or FUS, overexpressing TSLNC8 alone and in combination, inhibits the growth of transplanted tumours and prolongs survival time in nude mice

**Fig. 8:**
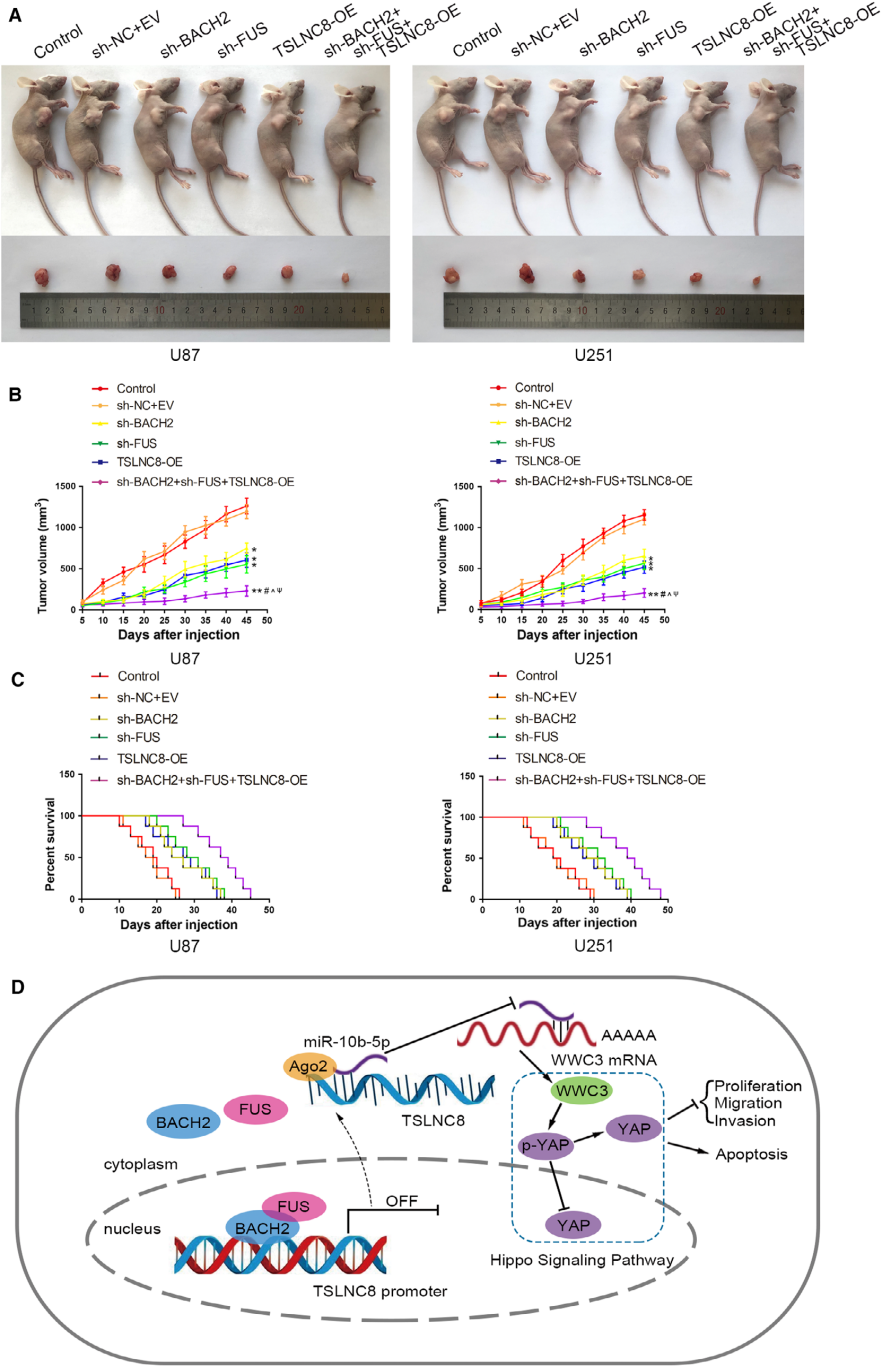
Tumour xenograft studies. (A) The nude mice carrying tumours from the respective groups are shown. The sample tumours excised from the respective groups are shown. (B) Tumour growth curves of six nude mice groups are shown, and data are presented as mean ± SD (*n* = 8 for each group). Tumour volume was calculated every 5 days after injection, and the tumour was excised after 45 days. Data are analysed by using one‐way ANOVA *P < 0.05, **P < 0.01 vs. control group; ^#^ *P* < 0.05 vs. sh‐BACH2 group; ^^^ *P* < 0.05 vs. sh‐FUS group; ^ψ^ *P* < 0.05 vs. TSLNC8‐OE group. (C) Survival curves of nude mice injected into the right striatum from the respective groups are shown (*n* = 8, each group). Data are analysed by using log‐rank test, *P* < 0.05 for sh‐BACH2, sh‐FUS or TSLNC8‐OE group vs. control group; *P* < 0.01 for sh‐BACH2+sh‐FUS+TSLNC8 group‐OE vs. control group. (D) Schematic drawing of the mechanism of the BACH2/TSLNC8/miR‐10b‐5p/wwc3 axis in glioma cells.

The functions of BACH2, FUS and TSLNC8 were further clarified by subcutaneous xenograft and orthotopic inoculation glioma models in nude mice. Compared to the control group, the sh‐BACH2, sh‐FUS, TSLNC8‐OE and sh‐BACH2+sh‐FUS+TSLNC8‐OE groups produced smaller tumour volumes. Additionally, the transplanted tumour in the sh‐BACH2+sh‐FUS+TSLNC8‐OE group was the smallest (Fig. 8A,B). The volume change data of subcutaneous xenograft is provided in Table S3. Survival analysis showed that the survival time of the sh‐BACH2, sh‐FUS, TSLNC8‐OE and sh‐BACH2+sh‐FUS+TSLNC8‐OE groups of nude mice was significantly prolonged. The sh‐BACH2+sh‐FUS+TSLNC8‐OE group had the longest survival time compared with the control group (Fig. 8C).

## 4 Discussion

The characterisation of the protein‒protein interactions in glioma may help to improve our understanding of the regulation of the malignant progression of glioma cells. BACH2 expression is increased in glioma tissues and cells. The downregulation of BACH2 significantly inhibits the viability, migration and invasive ability of glioma cells and promotes glioma cell apoptosis, suggesting that BACH2 may act as an oncogene. Chen *et al*. [32] found that BACH2 is upregulated in NPC, while its knockdown inhibits the proliferation, migration and invasion of NPC cells. BACH2 expression varies in different tumours; CD19+ B cells from mantle cell lymphoma patient samples showed reduced BACH2 levels. In addition, BACH2 is an epigenetic target for inhibiting the proliferation of gastric cancer cells [33].

Fused in sarcoma is dysregulated in a variety of tumours. We found that FUS was upregulated in glioma tissues and cells. The downregulation of FUS significantly inhibited the malignant behaviour of glioma cells, suggesting that FUS may act as an oncogene. Recently, FUS was reported to be upregulated in neuroblastoma cells, while the silencing of FUS was found to inhibit neuroblastoma cell proliferation and cell cycle transition [34]. Furthermore, the silencing of FUS was found to promote the apoptosis of breast cancer cells [35].

Interestingly, the interaction between BACH2 and FUS may represent a novel target for the treatment of glioma. In this study, the dual knockdown of BACH2 and FUS was found to exhibit stronger inhibition of the malignant behaviour of glioma cells, indicating that the interaction enhanced the cancer‐promoting effect. FUS directly affects transcriptional regulation and RNA processing as RNA‐binding proteins [36]. For example, FUS‐mediated alternative splicing in the nervous system affects the development of ALS and FTLD [37]. However, in the present study, FUS acted as a co‐activator and supported the regulation of downstream target genes by BACH2. Similarly, Haile *et al*. [38] demonstrated that FUS acts as a co‐activator, interacting with androgen receptors to facilitate the growth and survival of prostate cancer cells. In addition, studies have confirmed that FUS interacts with specific transcription factors, such as NF‐κB and MITF, to regulate gene expression [39, 40].

lncRNAs play a key role in the formation and progression of various tumours. Some studies have reported that SOX2OT, OIP5‐AS1 and UCA1 affect the biological behaviour of glioma cells [25, 28, 41]; the expression of SNHG1 is abnormal in rectal cancer, liver cancer, lung cancer and gastric cancer tumours, while the upregulation of SNHG1 is associated with tumour stage and size and a reduction in the overall survival rate [42]. Our study showed that TSLNC8 was downregulated in glioma tissues and cells. TSLNC8 overexpression significantly inhibited the viability, migration and invasion of glioma cells and promoted glioma cell apoptosis, indicating that TSLNC8 acts as a tumour suppressor in glioma cells, which is consistent with the findings reported by Chen and Yu in their study [18]. However, our findings further revealed the mechanism by which TSLNC8 regulates the biological behaviour of glioma cells. Furthermore, we found that the knockdown of BACH2 significantly upregulated TSLNC8 expression and that BACH2 negatively regulated TSLNC8 expression by binding to the promoter region of TSLNC8. In a previous study, BACH2 was reported to play a major role in transcriptional inhibition as a transcription factor [43], and related studies have shown that BACH2 binds to the promoter of IL‐2 in Jurkat T cells and inhibits IL‐2 transcription [44]. In the present study, we also found that the dual knockdown of BACH2 and FUS significantly enhanced the promotion expression of TSLNC8 induced by the knockdown of BACH2 or FUS alone. This demonstrated that the interaction between BACH2 and FUS enhanced the negative regulation of TSLNC8 by BACH2, thus facilitating the viability, migration and invasion of glioma cells and the inhibition of glioma cell apoptosis.

microRNAs (miRNAs) play important regulatory roles in cells, including the regulation of gene expression via the degradation or translational inhibition of target mRNAs [45]. In the present study, we confirmed that miR‐10b‐5p was highly expressed in glioma tissues and cells. miR‐10b‐5p overexpression significantly promoted the malignancy of glioma cells, while its inhibition had the opposite effect, suggesting that miR‐10b‐5p may act as a tumour promoter in glioma cells. Previous studies have reported that miR‐17 is highly expressed in colorectal cancer tissue, wherein the overexpression of miR‐17 facilitates the migration and invasion of colorectal cancer cells [46]. Six miRNAs, including miR‐10b‐5p, are significantly upregulated in glucocorticoid‐induced osteoporosis [47]. miR‐590‐3p targets CCNG2 and FOXO3 [48] to promote ovarian cancer development. miR‐10b‐5p has various physiological functions, and the overexpression of miR‐10b‐5p has been previously found to promote the proliferation of myoblast C2C12 cells and weaken muscle fibre formation [49]. We investigated the regulation of miR‐10b‐5p in U87 and U251 cells and demonstrated the mechanism of miR‐10b‐5p regulation of glioma cell behaviour. In the present study, we found that TSLNC8 and miR‐10b‐5p had opposite effects on the biological behaviour of glioma cells. In addition, TSLNC8 negatively regulated miR‐10b‐5p expression in a sequence‐dependent manner. The overexpression of miR‐10b‐5p reversed the inhibitory effect of TSLNC8 overexpression on glioma cell malignancy. Numerous studies have shown that lncRNAs negatively regulate miRNA expression in a sequence‐dependent manner. For example, TP73‐AS1 binds to miR‐449a and downregulates its expression in a RISC‐dependent manner to promote the development of non‐small cell lung cancer [50].

In recent years, the regulation of WWC3 in tumours has received a lot of attention. Some studies have reported that WWC3 inhibits the epithelial‒mesenchymal transition of lung cancer by activating the Hippo‐YAP signalling pathway [51]. The downregulation of WWC3 is associated with the inhibition of Hippo signalling and results in a poor prognosis of human gastric cancer [52]. In this study, the overexpression of WWC3 significantly restrained the viability, migration and invasion of glioma cells and promoted glioma cell apoptosis, while the knockdown of WWC3 had the opposite effect, suggesting that WWC3 acts as a tumour suppressor in glioma cells.

This study confirmed that the overexpression of WWC3 significantly upregulated the phosphorylation of YAP and reduced YAP influx into the nucleus, while the knockdown of WWC3 had the opposite effect. As a key upstream signalling molecule of the Hippo signalling pathway, WWC3 activated this signalling pathway and inhibited the malignancy of glioma cells. Hippo signalling pathways are involved in the regulation of cell proliferation, organ size control, stem cell function and tumour development [53, 54, 55]. It has been reported that LATS2 inhibits the growth of lung cancer cells by downregulating the cyclin E/CDK2 kinase activity [56]. YAP is significantly upregulated in gastric dysplasia, gastric adenocarcinoma and metastatic gastric cancer [57], while Cbx7 inhibits the migration of glioma cells by inhibiting the YAP/TAZ‐CTGF‐JNK signalling axis [58].

microRNAs inhibit downstream mRNA translation by targeting the 3′‐UTR region of the target mRNA. In this study, dual‐luciferase reporter gene analysis demonstrated that miR‐10b‐5p binds to the WWC3 3′‐UTR region, and the knockdown of miR‐10b‐5p upregulated the expression of WWC3 mRNA and protein. A similar study found that miR‐384 inhibited the malignant progression of glioma by inhibiting PIWIL4 expression by targeting its 3′‐UTR region [26], while miR‐331‐3p downregulated E2F1 expression and promoted the proliferation and migration of hepatoma cells [59]. lncRNAs are able to bind to miRNAs and act as a molecular sponge of competitive endogenous RNAs, inhibiting the negative regulation of target genes by miRNAs and regulating tumorigenesis and development. DLX6‐AS1 attenuates the inhibition of E2F1 through competitive binding to miR‐197‐5p, thereby accelerating the development of glioma [60]. SNHG7 promotes the proliferation of pancreatic cancer cells through ID4 by competitively binding miR‐342‐3p [61]. TSLNC8 serves as a competitive endogenous RNA, attenuating the miR‐10b‐5p inhibitory effect on WWC3, thereby inhibiting the viability, migration and invasion of glioma cells and promoting glioma cell apoptosis.

Finally, an *in vivo* study demonstrated that the knockdown of BACH2 or FUS, the overexpression of TSLNC8 or a combination of the three inhibited the growth of subcutaneous xenografts and prolonged the survival time in nude mice. The combination of the three resulted in the smallest xenograft and the longest survival. These results suggest that the knockdown of BACH2 or FUS, the overexpression of TSLNC8 and the combination of the three have potential for use as targets in the therapeutic treatment of glioma.

## 5 Conclusion

This study is the first to demonstrate that high levels of BACH2 expression enhance the transcriptional inhibition of TSLNC8 by interacting with FUS, resulting in an attenuation of the negative regulatory effect of TSLNC8 on miR‐10b‐5p. This was found to reduce WWC3 expression by enhancing the binding of miR‐10b‐5p to the WWC3 3′‐UTR region, blocking the Hippo signalling pathway and promoting the malignancy of glioma cells (Fig. 8D). Our findings provide insights into the mechanism underlying the development of glioma and provide a novel strategy for the development of glioma therapeutic treatments.

## Conflict of interest

The authors declare no conflict of interest.

## Author contributions

YL, YX and YY designed the research and drafted the manuscript. YY led the research and analysed the data. YY, XL and JZ performed the experiment and acquired the data. LL, JM and PW analysed the bioinformatic database and helped acquire data. CY, DW, LS and XR performed the experiments.

## Ethical approval and consent to participate

This study was approved by the Ethics Committee of Shengjing Hospital of China Medical University, and informed consent was obtained from all participants. All experiments with mice were conducted strictly in accordance with the Animal Welfare Act approved by the Ethics Committee of China Medical University. The study was performed in accordance with the Declaration of Helsinki.

## Supporting information


**Fig. S1.** The gene expression levels for transient or stable transfection. qRT‐PCR analysis of BACH2 expression in U87 and U251 cells after transient transfection. Data are presented as mean ± SD (*n* = 3 for each group), and analysed by using one‐way ANOVA. **P < 0.01 vs. sh‐NC group. (B) Western blotting assay was used to measure the expression of BACH2 in U87 and U251 cell safter stable transfection of BACH2 knockdown. Data are presented as mean ± SD (*n* = 3 for each group), and analysed by using one‐way ANOVA. **P < 0.01 vs. sh‐NC group. (C) qRT‐PCR analysis of FUS expression in U87 and U251 cells after transient transfection. Data are presented as mean ± SD (*n* = 3 for each group), and analysed by using one‐way ANOVA. **P < 0.01 vs. sh‐NC group. (D) Western blot assay was used to measure the expression of FUS in U87 and U251 cells after stable transfection of FUS knockdown. Data are presented as mean ± SD (*n* = 3 for each group), and analysed by using one‐way ANOVA. **P < 0.01 vs. sh‐NC group. (E) qRT‐PCR analysis of TSLNC8 expression in U87 and U251 cells after stable transfection. Data are presented as mean ± SD (*n* = 3 for each group), and analysed by using one‐way ANOVA. **P < 0.01 vs. EV group. (F, G) qRT‐PCR analysis of miR‐10b‐5p expression in U87 and U251 cells after transient transfection. Data are presented as mean ± SD (*n* = 3 for each group), and analysed by using one‐way ANOVA. **P < 0.01 vs. agomiR‐10b‐5p ‐NC group; **P < 0.01 vs. antagomiR‐10b‐5p‐NC group. (H) qRT‐PCR analysis of WWC3 expression in U87 and U251 cells after stable transfection. Data are presented as mean ± SD (*n* = 3 for each group), and analysed by using one‐way ANOVA. **P < 0.01 vs. EV group. (I) Western blotting assay was used to measure the expression of WWC3 in U87 and U251 cells after stable transfection of WWC3 overexpression. Data are presented as mean ± SD (*n* = 3 for each group), and analysed by using one‐way ANOVA. **P < 0.01 vs. EV group. (J) qRT‐PCR analysis of WWC3 expression in U87 and U251 cells after transient transfection. Data are presented as mean ± SD (*n* = 3 for each group), and analysed by using one‐way ANOVA. **P < 0.01 vs. sh‐NC group. (K) Western blotting assay was used to measure the expression of WWC3 in U87 and U251 cells after stable transfection of WWC3 knockdown. Data are presented as mean ± SD (*n* = 3 for each group), and analysed by using one‐way ANOVA. **P < 0.01 vs. sh‐NC group. (L) qRT‐PCR analysis of BACH2 expression in U87 and U251 cells after lentiviral stable transfection of BACH2 knockdown. Data are presented as mean ± SD (*n* = 3 for each group), and analysed by using one‐way ANOVA. **P < 0.01 vs. sh‐NC group. (M) qRT‐PCR analysis of FUS expression in U87 and U251 cells after lentiviral stable transfection of FUS knockdown. Data are presented as mean ± SD (*n* = 3 for each group), and analysed by using one‐way ANOVA. **P < 0.01 vs. sh‐NC group. (N) qRT‐PCR analysis of TSLNC8 expression in U87 and U251 cells after lentiviral stable transfection of TSLNC8 overexpression. Data are presented as mean ± SD (*n* = 3 for each group), and analysed by using one‐way ANOVA. **P < 0.01 vs. EV group.

**Fig. S2.** Screening of genes of interest and the effects of TSLNC8 on the biological behaviour of glioma cells. (A) BACH2 expression levels in the GSE database. (B) FUS expression levels in the GSE database. (C) TSLNC8 gene expression profiles in U87 and u251 cells (*n* = 3). (D) qRT‐PCR analysis of the selected molecules. Data are presented as mean ± SD (*n* = 3 for each group), and analysed by using two‐way ANOVA. *P < 0.05, **P < 0.01 vs. sh‐NC group. (E) MiR‐10b‐5p gene expression profiles in U87 and u251 cells (*n* = 3). (F) Validation of qRT‐PCR analysis of the selected molecules. Data are presented as the mean ± SD (*n* = 3 for each group), and analysed by using two‐way ANOVA. *P < 0.05, **P < 0.01 vs. EV group. (G) qRT‐PCR analysis of TSLNC8 expressions in U87 and U251 cells after transient knockdown of TSLNC8. Data are presented as mean ± SD (*n* = 3 for each group), and analysed by using one‐way ANOVA. **P < 0.01 vs. sh‐NC group. (H) CCK‐8 assay was used to measure the effect of TSLNC8 on the viability of U87 and U251 cells. (I) Transwell assays were used to measure the effect of TSLNC8 on cell migration and invasion of U87 and U251 cells. (J) Flow cytometry analysis of U87 and U251 cells treated with altered expression of TSLNC8. (H‐J) Data are presented as mean ± SD (*n* = 3 for each group), and analysed by using one‐way ANOVA. *P < 0.05, **P < 0.01 vs sh‐NC group. Scale bar represents 20 μm. (K) Western blotting assay was used to measure the cytoplasmic YAP expression in U87 and U251 cells treated with WWC3 overexpression or knockdown. Data are presented as mean ± SD (*n* = 3 for each group), and analysed by using one‐way ANOVA. **P < 0.01 vs. EV group; ^#^ *P* < 0.05 vs. sh‐NC group. (L) Western blotting assay was used to measure the nuclear YAP expression in U87 and U251 cells treated with WWC3 overexpression or knockdown. Data are presented as mean ± SD (*n* = 3 for each group), and analysed by using one‐way ANOVA. *P < 0.05 vs. EV group; ^##^ *P* < 0.01 vs. sh‐ NC group. Scale bar represents 40 μm.

**Table S1**. The clinical information of donors and patients

**Table S2.** The primers of BACH2 mRNA, FUS mRNA, TSLNC8, miR10b‐5p, WWC3 mRNA in Quantitative real‐time PCR (qRT‐PCR).

**Table S3.** Raw data of the subcutaneous xenograft.

## Data Availability

The data and materials associated with the current study are available from the corresponding author upon reasonable request.
