# Supplementary figures and images for: Interaction of BACH2 with FUS promotes malignant progression of glioma cells via the TSLNC8–miR‐10b‐5p–WWC3 pathway
Source: Mol Oncol. 2020 Sep 26;14(11):2936–59. doi: 10.1002/1878-0261.12795 (PMC7607167; doi:10.1002/1878-0261.12795)

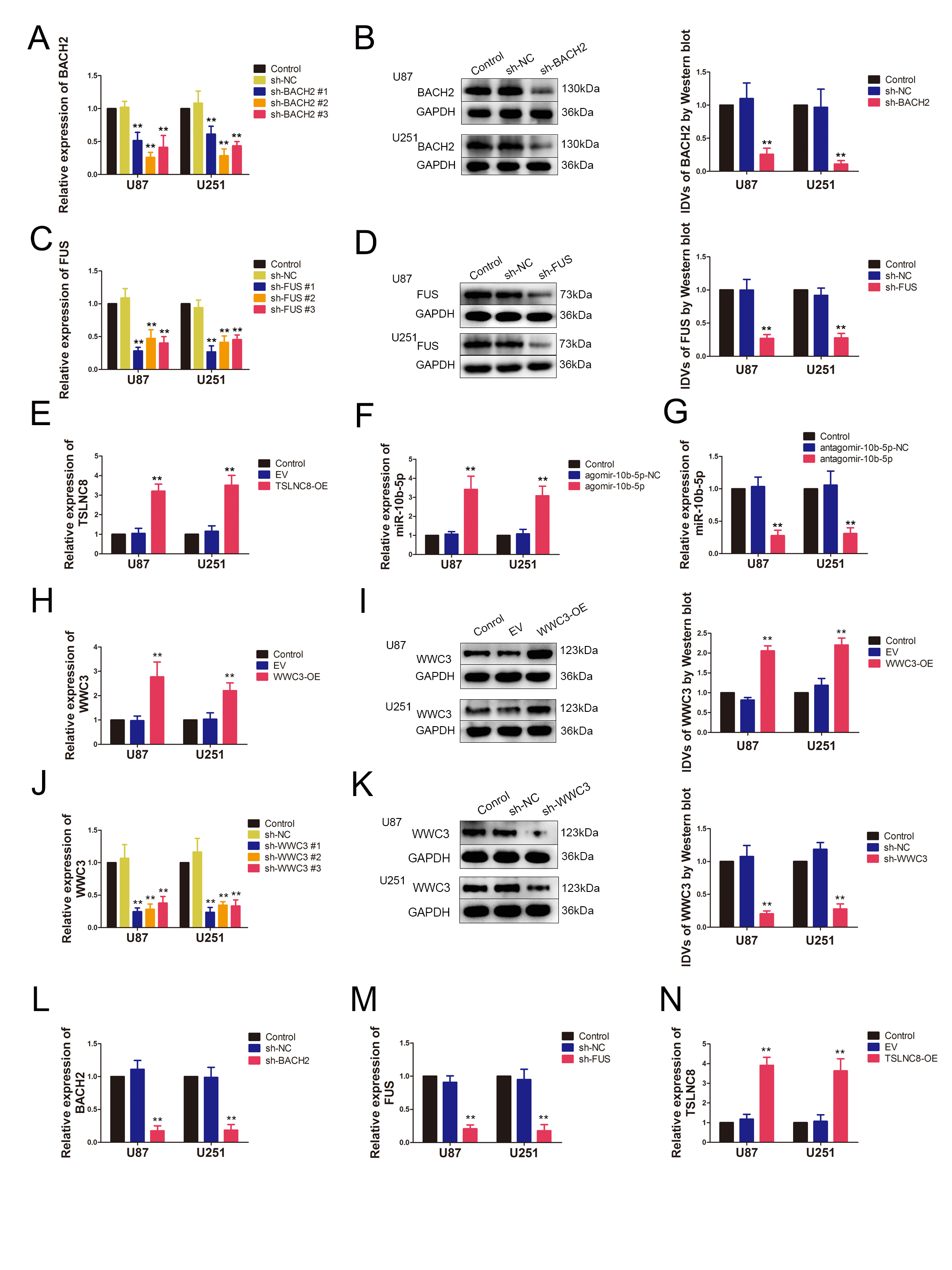

Supplement: Supplementary file 1 — Fig. S1. The gene expression levels for transient or stable transfection. qRT‐PCR analysis of BACH2 expression in U87 and U251 cells after transient transfection. Data are presented as mean ± SD (n = 3 for each group), and analysed by using one‐way ANOVA. **P < 0.01 vs. sh‐NC group. (B) Western blotting assay was used to measure the expression of BACH2 in U87 and U251 cell safter stable transfection of BACH2 knockdown. Data are presented as mean ± SD (n = 3 for each group), and analysed by using one‐way ANOVA. **P < 0.01 vs. sh‐NC group. (C) qRT‐PCR analysis of FUS expression in U87 and U251 cells after transient transfection. Data are presented as mean ± SD (n = 3 for each group), and analysed by using one‐way ANOVA. **P < 0.01 vs. sh‐NC group. (D) Western blot assay was used to measure the expression of FUS in U87 and U251 cells after stable transfection of FUS knockdown. Data are presented as mean ± SD (n = 3 for each group), and analysed by using one‐way ANOVA. **P < 0.01 vs. sh‐NC group. (E) qRT‐PCR analysis of TSLNC8 expression in U87 and U251 cells after stable transfection. Data are presented as mean ± SD (n = 3 for each group), and analysed by using one‐way ANOVA. **P < 0.01 vs. EV group. (F, G) qRT‐PCR analysis of miR‐10b‐5p expression in U87 and U251 cells after transient transfection. Data are presented as mean ± SD (n = 3 for each group), and analysed by using one‐way ANOVA. **P < 0.01 vs. agomiR‐10b‐5p ‐NC group; **P < 0.01 vs. antagomiR‐10b‐5p‐NC group. (H) qRT‐PCR analysis of WWC3 expression in U87 and U251 cells after stable transfection. Data are presented as mean ± SD (n = 3 for each group), and analysed by using one‐way ANOVA. **P < 0.01 vs. EV group. (I) Western blotting assay was used to measure the expression of WWC3 in U87 and U251 cells after stable transfection of WWC3 overexpression. Data are presented as mean ± SD (n = 3 for each group), and analysed by using one‐way ANOVA. **P < 0.01 vs. EV group. (J) qRT‐PCR analysis of WWC3 expression [file MOL2-14-2936-s001.tif]

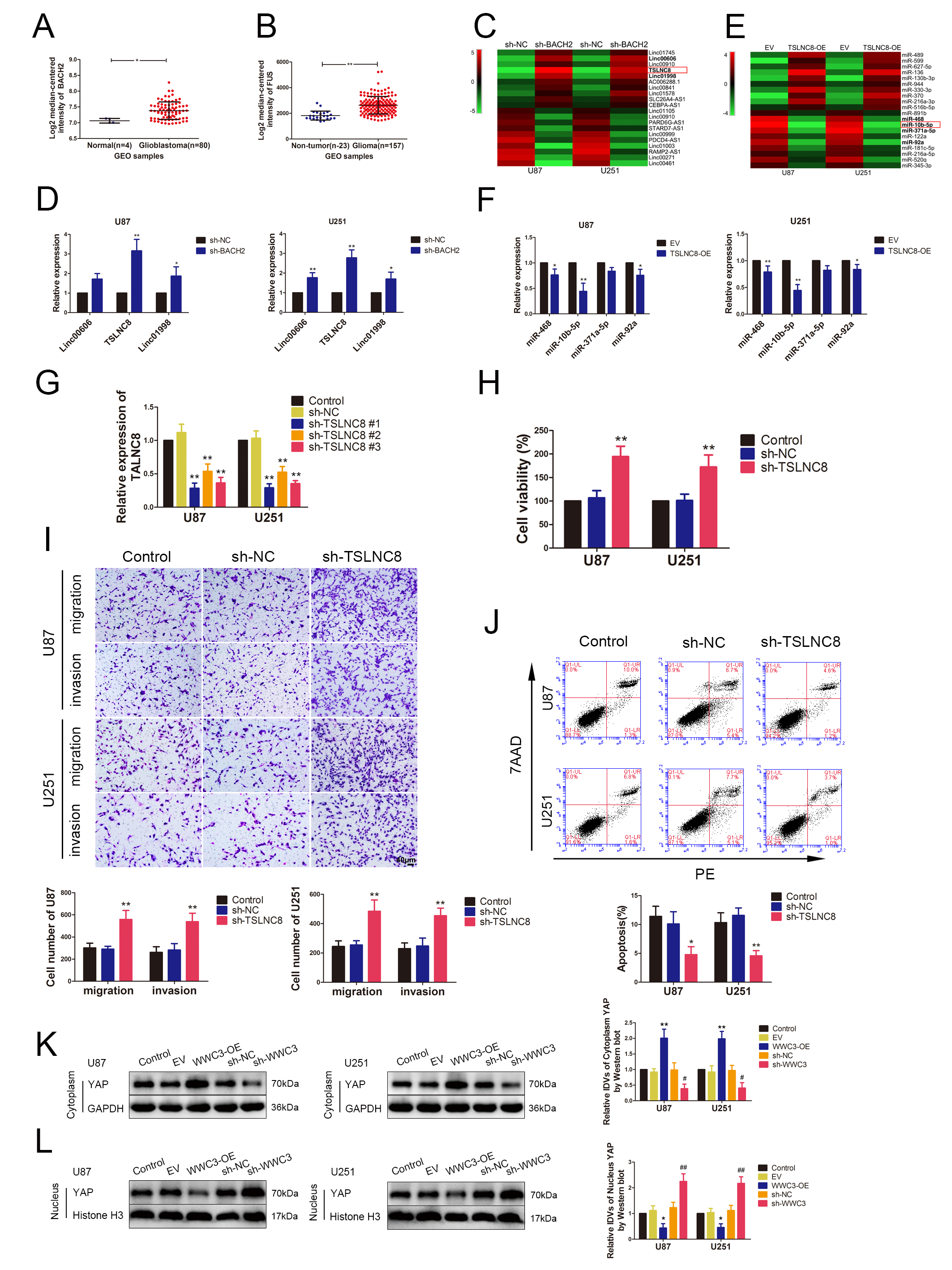

Supplement: Supplementary file 2 — Fig. S2. Screening of genes of interest and the effects of TSLNC8 on the biological behaviour of glioma cells. (A) BACH2 expression levels in the GSE database. (B) FUS expression levels in the GSE database. (C) TSLNC8 gene expression profiles in U87 and u251 cells (n = 3). (D) qRT‐PCR analysis of the selected molecules. Data are presented as mean ± SD (n = 3 for each group), and analysed by using two‐way ANOVA. *P < 0.05, **P < 0.01 vs. sh‐NC group. (E) MiR‐10b‐5p gene expression profiles in U87 and u251 cells (n = 3). (F) Validation of qRT‐PCR analysis of the selected molecules. Data are presented as the mean ± SD (n = 3 for each group), and analysed by using two‐way ANOVA. *P < 0.05, **P < 0.01 vs. EV group. (G) qRT‐PCR analysis of TSLNC8 expressions in U87 and U251 cells after transient knockdown of TSLNC8. Data are presented as mean ± SD (n = 3 for each group), and analysed by using one‐way ANOVA. **P < 0.01 vs. sh‐NC group. (H) CCK‐8 assay was used to measure the effect of TSLNC8 on the viability of U87 and U251 cells. (I) Transwell assays were used to measure the effect of TSLNC8 on cell migration and invasion of U87 and U251 cells. (J) Flow cytometry analysis of U87 and U251 cells treated with altered expression of TSLNC8. (H‐J) Data are presented as mean ± SD (n = 3 for each group), and analysed by using one‐way ANOVA. *P < 0.05, **P < 0.01 vs sh‐NC group. Scale bar represents 20 μm. (K) Western blotting assay was used to measure the cytoplasmic YAP expression in U87 and U251 cells treated with WWC3 overexpression or knockdown. Data are presented as mean ± SD (n = 3 for each group), and analysed by using one‐way ANOVA. **P < 0.01 vs. EV group; # P < 0.05 vs. sh‐NC group. (L) Western blotting assay was used to measure the nuclear YAP expression in U87 and U251 cells treated with WWC3 overexpression or knockdown. Data are presented as mean ± SD (n = 3 for each group), and analysed by using one‐way ANOVA. *P < 0.05 vs. EV group; ## P < 0.01 vs. sh‐ NC group. Sc [file MOL2-14-2936-s002.tif]
